# MDIG-mediated H3K9me3 demethylation upregulates Myc by activating OTX2 and facilitates liver regeneration

**DOI:** 10.1038/s41392-023-01575-5

**Published:** 2023-09-15

**Authors:** Jinpeng Du, Wenwei Liao, Haichuan Wang, Guimin Hou, Min Liao, Lin Xu, Jiwei Huang, Kefei Yuan, Xiangzheng Chen, Yong Zeng

**Affiliations:** 1 Division of Liver Surgery, Department of General Surgery and Laboratory of Liver Surgery, and State Key Laboratory of Biotherapy and Collaborative Innovation Center of Biotherapy, West China Hospital, Sichuan University, Chengdu, Sichuan 610041 China; 2 Department of Hepatobiliary and Pancreatic Surgery, The First Affiliated Hospital of Zhengzhou University, Zhengzhou, Henan 450052 China; 3 Department of Thoracic Surgery, The Second Clinical Medical College, Jinan University (Shenzhen People’s Hospital), Shenzhen, 518020 China; 4 The First Affiliated Hospital, Jinan University, Guangzhou, Guangdong 510630 China; 5 Department of Hepato-Biliary-Pancreatic Surgery, Sichuan Cancer Hospital & Institute, Sichuan Cancer Center, The Affiliated Cancer Hospital of University of Electronic Science and Technology of China, Chengdu, Sichuan 610041 China; 6 Department of Medical Ultrasound, West China Hospital, Sichuan University, Chengdu, Sichuan 610041 China

**Keywords:** Epigenetics, Epigenetics

## Abstract

The mineral dust-induced gene (MDIG) comprises a conserved JmjC domain and has the ability to demethylate histone H3 lysine 9 trimethylation (H3K9me3). Previous studies have indicated the significance of MDIG in promoting cell proliferation by modulating cell-cycle transition. However, its involvement in liver regeneration has not been extensively investigated. In this study, we generated mice with liver-specific knockout of MDIG and applied partial hepatectomy or carbon tetrachloride mouse models to investigate the biological contribution of MDIG in liver regeneration. The MDIG levels showed initial upregulation followed by downregulation as the recovery progressed. Genetic MDIG deficiency resulted in dramatically impaired liver regeneration and delayed cell cycle progression. However, the MDIG-deleted liver was eventually restored over a long latency. RNA-seq analysis revealed Myc as a crucial effector downstream of MDIG. However, ATAC-seq identified the reduced chromatin accessibility of OTX2 locus in MDIG-ablated regenerating liver, with unaltered chromatin accessibility of Myc locus. Mechanistically, MDIG altered chromatin accessibility to allow transcription by demethylating H3K9me3 at the OTX2 promoter region. As a consequence, the transcription factor OTX2 binding at the Myc promoter region was decreased in MDIG-deficient hepatocytes, which in turn repressed Myc expression. Reciprocally, Myc enhanced MDIG expression by regulating MDIG promoter activity, forming a positive feedback loop to sustain hepatocyte proliferation. Altogether, our results prove the essential role of MDIG in facilitating liver regeneration via regulating histone methylation to alter chromatin accessibility and provide valuable insights into the epi-transcriptomic regulation during liver regeneration.

## Introduction

The mammalian liver possesses a remarkable capacity to successfully restore the liver mass and function to pre-injury status within a few weeks, which is generally referred to as liver regeneration. Upon stimulation, the residual quiescent hepatocytes re-enter the cell cycle and initiate mitosis at a high rate to generate new cells.^1,2^ Multiple signaling molecules regulate cell proliferation during liver regeneration. It has been reported that over 100 genes in hepatocytes are activated within 1 h after partial hepatectomy (PH), and this episodic alteration of gene expression is sustained for more than 14 days until complete reconstruction of the normal hepatic architecture.^3,4^ Under the precise modulation of genetic and epigenetic genes, the regenerative activities of hepatocytes are kept to be phenotypic fidelity. Nonetheless, aberrant regulation of this process can cause failure of regeneration, and the development of liver cirrhosis and hepatocellular carcinoma (HCC).^5,6^ Hence, understanding the mechanisms that govern the switch between quiescence and regeneration of adult hepatocytes will have important implications for identifying therapeutic targets of human liver disease.

Eukaryotic DNA is highly organized and structured into transcriptionally silent heterochromatin and active euchromatin.^7^ The active regions of euchromatin characterized by histone modifications maintain access to transcription machinery components and activate gene transcription. However, heterochromatin marked by histone and DNA methylation impairs the accessibility of promoters to transcription factors, resulting in gene silencing.^8–10^ In mammals, histone methylation is an important epigenetic modification associated with gene repression at the promoter regions.^11^ The histone can be mono-, di- and trimethylated in its lysine or ε-amine group. For example, trimethylation of Lys 9 of histone 3 (H3K9me3) has been identified as a repressive modification, and trimethylation of Lys 4 of histone 3 (H3K4me3) as activating mark.^12^ H3K9me3 is mainly catalyzed by histone methyltransferase Suv39h1 and can be erased by Jumonji family proteins. By remaining compact heterochromatin conformation, H3K9me3 can be enriched in the transcription start sites (TSS) of repressed genes, and it is related to transcriptional inhibition.^13,14^ For instance, reduced H3K9me3 increases chromatin accessibility of most genes involved in cell growth and cell cycle transition, causing tumor initiation and progression.^15,16^ In addition, demethylation of H3K9me3 in LINE-1 leads to increased rates of chromosome missegregation and disrupts genome stability, which results in tumorigenesis of breast cancer.^17^ Thus, dysregulation of histone methylation affects gene expression, which then induces aberrant biological progress. Furthermore, H3K27me3 emerged as an essential component of the epigenetic code that governs genes to be differentially expressed during liver regeneration. However, the role of H3K9me3 during liver regeneration, which is highly involved in the dynamic and fast regulation of lots of genes, has not been clarified yet.

The mineral dust-induced gene (MDIG) was first identified in alveolar macrophages from miners who have been exposed to mineral dust.^18^ Independently, this gene is also discovered as a nuclear protein transcriptionally regulated by Myc in human promyelocytic leukemia HL60 cells and hence is named Myc-induced nuclear antigen 53 (mina53).^19^ In addition, the MDIG is reported to demethylate H3K9me3 to the monomethylated state through a JmjC domain within its amino acid residue, a signature motif in most histone demethylases.^20^ MDIG seems to act as an oncogene as its upregulation has been frequently found to be associated with the malignancy of a number of tumors, such as glioblastoma, breast cancer, and HCC.^15,20–22^ Up to 70% of HCC samples collected from 155 patients showed the overexpression of MDIG. Silencing of MDIG in HCC cell lines significantly promoted the enrichment of H3K9me3 at the promoter regions of CDC6, which in turn led to decreased CDC6 expression and repressive cell-cycle progression.^16^ Furthermore, emerging evidence demonstrates that epigenetic code is embedded in quiescent tissue to dictate the gene expression pattern required for regeneration.^23^ Thus, it is plausible that as an epigenetic modifier acting on histone, MDIG may be involved in regulating liver regeneration, which warrants our investigation.

In this study, we identified MDIG as a critical epigenetic factor for liver regeneration. We observed a rapid increase in MDIG expression accompanied by a significant reduction in total H3K9me3 modification after PH. Liver-specific deletion of MDIG significantly impaired liver regeneration following 70% PH or CCl_4_ challenge. Mechanistically, the regenerating MDIG-deficient hepatocytes displayed retarded cell growth and arrested cell cycle at least partly via regulation of orthodenticle homeobox 2 (OTX2) promoter region. The OTX2 encodes a member of the bicoid subfamily of homeodomain-containing transcription factors that influences the proliferation and differentiation of dopaminergic neuronal progenitor cells during mitosis.^24^ Here, we reported that MDIG deletion reduced the chromatin accessibility of OTX2, which repressed Myc expression and thereby inhibited hepatic regenerative biological output.

## Results

### MDIG depletion impairs liver regeneration following a 70% PH or CCl_4_ challenge

To investigate the expression pattern of MDIG during liver regeneration, we collected liver tissues at different time points after 70% PH in mice. Western blot results displayed that the expression levels of MDIG markedly increased and rapidly peaked at 24 h post-hepatectomy, and then returned to normal levels slowly. Of note, the liver did not fully recover its mass until 168 h after PH. Correspondingly, a rapid decrease of trimethylation of H3K9 (H3K9me3) followed by a gradual increase was observed with the progress of regeneration, without any changes in mono- or di-methylation of H3K9 (H3K9me1 and H3K9me2) (Supplementary Fig. S1a, b). Consistent results were also found in the tissues collected from the carbon tetrachloride (CCl_4_) liver injury models (Supplementary Fig. S1c). These results indicated that by modulating histone methylation, MDIG may positively contribute to liver regeneration.

To elucidate the role of MDIG during liver regeneration, we generated liver-specific MDIG knockout mice (MDIG-KO) by alb-Cre/loxp system (Supplementary Fig. S1d).^25^ Of note, the MDIG-KO mice were born at Mendelian ratio and developed normally. No obvious dysfunction or morphologic abnormality was found during the time of observation (Supplementary Fig. S1e). Deletion of MDIG significantly increased total H3K9me3 levels in the quiescent hepatocytes (Supplementary Fig. S1f).

**Fig. 1:**
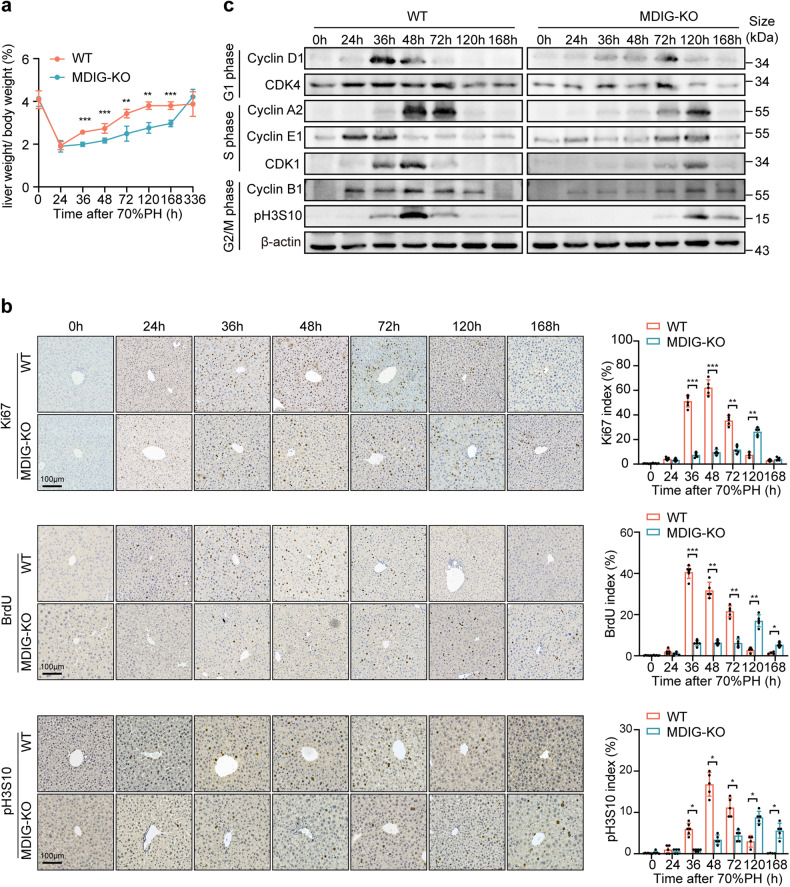
MDIG depletion impairs liver regeneration following 70% PH. **a** The ratios of liver weight/body weight at different time points after PH. **b** Immunohistochemistry results for Ki67, BrdU, and pH3S10 at different times following PH. **c** Western blot analysis of cell cycle markers at different times after PH in WT and MDIG-KO mice. Data were shown as mean ± SD, *n* = 4–6, unpaired Student’s *t*-test, *P < 0.05; **P < 0.01; ***P < 0.001. Scale bars, 100 μm

Next, we performed a 70% PH surgery on MDIG-KO mice and their control littermates (WT). To our surprise, MDIG ablation significantly impaired liver repair, as indicated by a much lower ratio of liver weight/body weight (LW/BW) along the observation (Fig. 1a). The MDIG-KO mice showed sustained higher levels of serum alanine transaminase (ALT), aspartate transaminase (AST) and total bilirubin (TBIL) than the WT mice (Supplementary Fig. S1g). As Ki67 and bromodeoxyuridine (BrdU) are specifically expressed at the mid-G1 and S phase of the cell cycle,^26^ respectively, fewer proliferating cells were demonstrated by immunohistochemistry (IHC) staining of Ki67 and BrdU at 0h- 72 h after PH. In addition, phospho-histone H3S10 (pH3S10), a protein expressed in the G2/M phase,^27^ was also significantly reduced in the MDIG-KO liver. However, more proliferating cells were observed at 120 and 168 h after PH in the MDIG-deleted livers (Fig. 1b). Eventually, the ratio of LW/BW and the liver function was restored in the MDIG-KO mice at fourteen days after PH (Fig. 1a and Supplementary Fig. S2a). The results suggest the knocking out of MDIG delayed proliferating responses during liver recovery.

Consistently, the expression levels of Cyclin D1 and CDK4, two markers of G1-phase, were markedly repressed in the MDIG-deficient liver; the expression of Cyclin E1 and Cyclin A2, both of which contributed to the G1/S transition,^28,29^ were correspondingly lower in the mutant livers. Additionally, the expression of Cyclin B1, CDK1, and pH3S10, which were specially expressed in the G2/M phase, were also obviously reduced in the MDIG-KO mice (Fig. 1c and Supplementary Fig. S2b, c). Together, MDIG ablation induced profoundly delayed cell cycle progression of hepatocytes during liver regeneration. In contrast, we applied adeno-associated virus (AAV) packaging MDIG (AAV-MDIG), with AAV-EGFP as the control, in the *C57BL/6J* mice to investigate the outcomes of MDIG overexpression on liver recovery. Excitingly, overexpression of MDIG promoted liver regeneration, as revealed by the intense increase in the LW/BW ratios from 24 h after PH. The LW/BW, as well as levels of liver enzymes, demonstrated significant differences from 36 h after PH until the end of observations between the MDIG overexpressed livers and the control livers. As expected, MDIG overexpression promoted cell proliferation as indicated by the increased index scores of Ki67, BrdU, and pH3S10 IHC stainings (Supplementary Fig. S3).

**Fig. 2:**
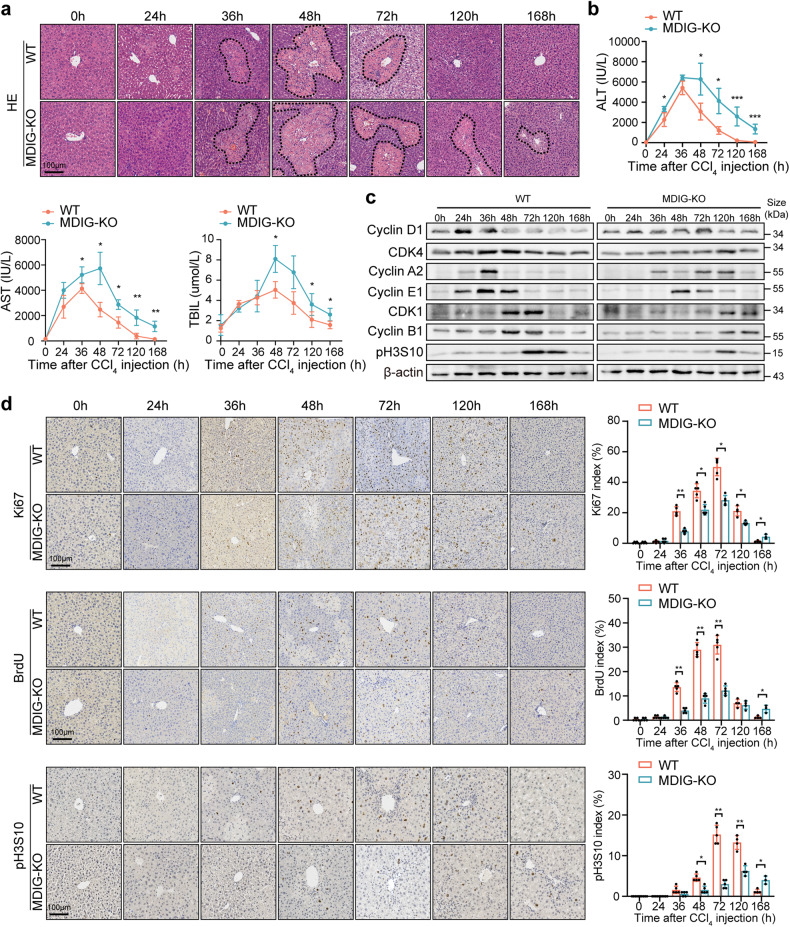
MDIG depletion impairs liver repair following the CCl_4_ challenge**. a** H&E staining showed liver repair was dramatically impaired in MDIG-KO liver. Necrotic areas were circled with dotted lines. **b** Serum ALT, AST, and TBIL levels at the indicated time points after the CCl_4_ challenge. **c** Western blot analysis of cell cycle markers at different times following the CCl_4_ challenge. **d** Immunohistochemistry results for Ki67, BrdU, and pH3S10 at different times following CCl_4_ challenge. Data were shown as mean ± SD, *n* = 3–5, unpaired Student’s *t*-test, *P < 0.05; **P < 0.01; ***P < 0.001. Scale bars, 100 μm

Subsequently, intraperitoneal injection of CCl_4_ into the mice was carried out to establish a liver injury model. Liver damage was noticeable in both the WT and MDIG-KO livers between 36 and 72 h after CCl_4_ injection, with the latter ones more severe. The livers of WT mice were completely repaired with undetectable necrosis by 120 h, while apparent necrotic hepatocytes were observed in the MDIG-KO mice until 168 h after CCl_4_ injection (Fig. 2a and Supplementary Fig. S4a). The MDIG-KO mice showed sustained higher levels of serum ALT, AST, and TBIL than the WT mice (Fig. 2c). Similar to the findings obtained from the PH model, the expression levels of cell cycle markers were significantly inhibited in the MDIG-KO mice, together with the reduced Ki67, BrdU, and pH3S10 indexes (Fig. 2d, e and Supplementary Fig. S4b, c).

Altogether, the current results suggest that MDIG deletion leads to delayed cell cycle progression and impairs liver regeneration in response to hepatic injury.

### MDIG deletion inhibits cell cycle progression in vitro

To further determine the functions of MDIG in cell proliferation, MDIG was knocked down in the Hepa1-6 cell line using small interfering RNAs (siRNAs) (Supplementary Fig. S5a). MDIG knockdown significantly inhibited cell proliferation, as demonstrated by lower CCK8 values, fewer colony formation numbers, and less EdU incorporation index (Supplementary Fig. S5b–d). In addition, lack of MDIG induced a significant inhibition of cell cycle progression, as flow cytometric results displayed obviously increased G1/G0 phase and decreased S phase and G2/M phase after silencing MDIG (Supplementary Fig. S5e). Conversely, overexpression of MDIG remarkably promoted cell proliferation and cell cycle progression (Supplementary Fig. S5f–j). Consistently, in the AML12 murine normal hepatocyte cell line, knocking down of MDIG resulted in suppression of cell proliferation while overexpression of MDIG promoted cell growth (Supplementary Fig. S6a–g).

In agreement with the results obtained in vivo, MDIG knockdown in vitro indeed repressed the expression of cell cycle proteins, while overexpression of MDIG upregulated these markers. As a demethylase of histone, MDIG could reduce the levels of H3K9me3, without any influences in H3K9me1 and H3K9me2 (Supplementary Fig. S6h).

### Loss of MDIG impairs liver regeneration by suppressing Myc expression

To further analyze the molecular alterations that occurred in response to MDIG deficiency during liver regeneration, we applied RNA-seq on the wildtype and MDIG-KO liver samples (Accession number: CRA007599). Of note, we reasoned that the regulatory activities mediated by the epigenetic modifier MDIG should occur at the time point following MDIG upregulation. Moreover, the regenerating activity in hepatocytes, as estimated by DNA synthesis, reaches its peak in the mouse at 36 h after 70% PH, according to a previous study.^30^ Therefore, we conducted gene expression profiling of livers at 0 h and 36 h after PH. The principal component analysis suggested slight variations among samples within each group but significant genetic dissimilarities among the four groups (Supplementary Fig. S7a). Altogether, the absence of MDIG induced 2599 gene alterations in the quiescent liver tissue, indicating a fundamental role of MDIG in diverse physiological processes (Supplementary Fig. S7b and Supplementary Table S1).

**Fig. 3:**
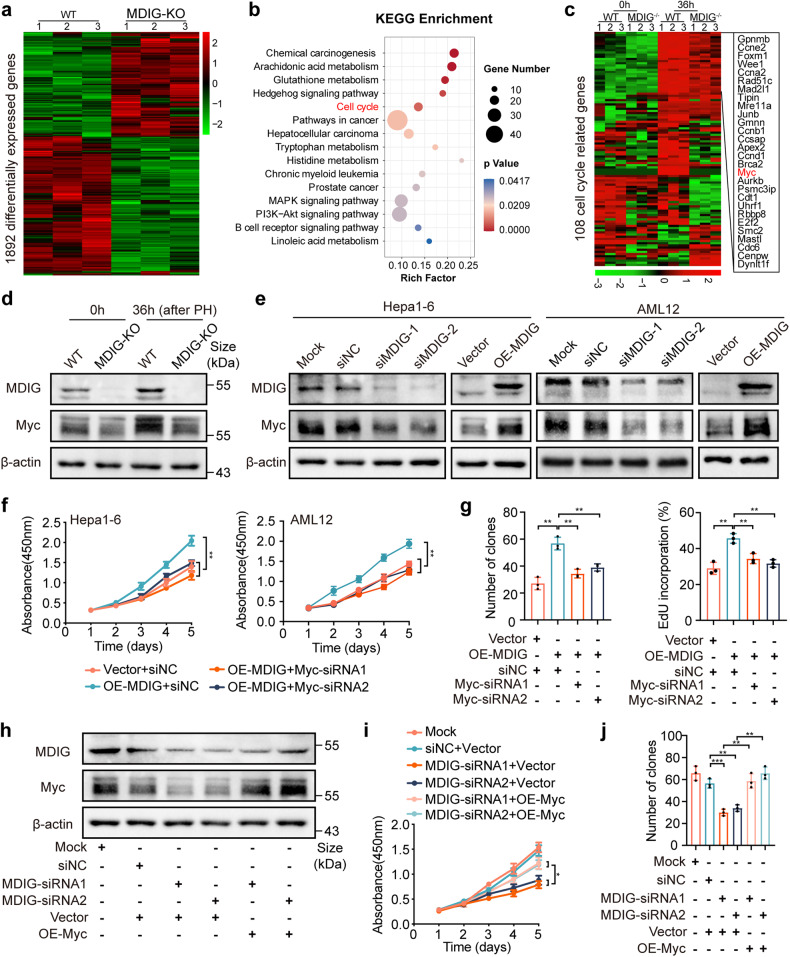
Loss of MDIG impairs liver regeneration by suppressing Myc expression. **a** A heat map of 1892 differentially expressed genes in WT and mutant livers at 36 h after PH. **b** KEGG pathway analysis of the primary altered genes indicated that the cell cycle pathway was significantly enriched during liver regeneration in the WT and MDIG-KO mice after 36 h PH. **c** Heat map of cell cycle-related genes from RNA-seq analysis of WT and MDIG-KO mice. The color bar showed the expression intensity. **d** Western blot for the indicated proteins in liver tissue lysates prepared from WT and MDIG-KO mice at 0 and 36 h after PH. **e** Western blot for the protein expression of Myc after MDIG knockdown or overexpression in Hepa1-6 and AML12 cells. **f** CCK8 assays revealed cell proliferation capacity after silencing Myc in MDIG-overexpressing Hepa1-6 and AML12 cells. **g** Colony formation assays (left) and EdU immunofluorescence assays (right) revealed cell proliferation capacity after silencing Myc in MDIG-overexpressing Hepa1-6 cells. **h** Western blot analysis showing the expression of MDIG and Myc after overexpressing Myc in MDIG-silencing Hepa1-6 cells. **i** CCK8 assays revealed cell proliferation capacity after overexpressing Myc in MDIG-silencing Hepa1-6 cells. **j** Colony formation assays revealed colony formation capacity after overexpressing Myc in MDIG-silencing Hepa1-6 cells. Data were shown as mean ± SD, unpaired Student’s *t*-test, *P < 0.05; **P < 0.01; ***P < 0.001

A total of 1892 genes were differentially expressed in MDIG-KO mice compared to WT mice at 36 h after PH, including 834 upregulated and 1058 downregulated genes (Fig. 3a and Supplementary Table S2). The KEGG pathway analysis of these deregulated genes showed that the cell cycle was significantly enriched. In addition, the MAPK and the PI3K-AKT signaling pathways, which were also important molecular cues during liver regeneration,^31,32^ were also enriched among the differentially expressed genes (Fig. 3b). The KEGG analysis further confirmed the important involvement of MDIG in multiple biological processes. As cell proliferation is essential for regenerative tissues, we further analyzed the crosstalk between MDIG and cell cycle genes. Notably, we identified 108 genes involved in cell cycle progression, and levels of Cyclin A2, Cyclin B1, Cyclin D1, and CDC6 were obviously decreased in the MDIG-KO mice at 36 h after surgery (Fig. 3c), which was consistent with proliferation defects observed in the mutant livers. Among these genes, Myc attracted our attention because of its obvious change and its crucial regulatory function in cell-cycle progression.^33^ Specifically, Myc was the most upregulated gene in the control livers at 36 h after PH compared to the 0 h samples, suggesting its important role during liver regeneration. In addition, levels of Myc were consistently lower in the MDIG-KO group than in the control group at both time points, suggesting its dependency on the presence of MDIG (Supplementary Table S3). Interestingly, Myc was reported to promote G1-S transition by controlling the activities of complexes Cyclin D-CDK4 and Cyclin E-CDK2 complexes.^34^ Furthermore, immunoblotting results demonstrated that Myc levels were remarkedly decreased in the MDIG-KO mice, regardless of whether they were quiescent or proliferating (Fig. 3d and Supplementary Fig. S7c). In contrast, other transcriptionally deregulated genes showed little difference at the protein levels between the WT and MDIG-KO groups (Supplementary Fig. S7d).

In agreement with these results, MDIG knockdown in vitro indeed repressed Myc expression, while overexpression of MDIG led to a marked increase of Myc expression (Fig. 3e). Then, we investigated whether the biological effects of MDIG were dependent on Myc. As expected, overexpression of Myc enhanced cell proliferation and clonogenicity, while silencing of Myc caused opposite results (Supplementary Fig. S8a–c). Flow cytometry results revealed that Myc promoted the G1-S progression (Supplementary Fig. S8d). Furthermore, the knockdown of Myc abolished the promoting effects of MDIG overexpression on cell proliferation, whereas the reintroduction of Myc significantly erased the suppressive effects mediated by MDIG knockdown on cell proliferation (Fig. 3e–j and Supplementary Fig. S9).

In summary, the data reveal Myc as a critical downstream effector of MDIG in regulating cell growth during liver regeneration.

### MDIG ablation reduces chromatin accessibility for OTX2 by increasing the H3K9 methylation of its promoter

**Fig. 4:**
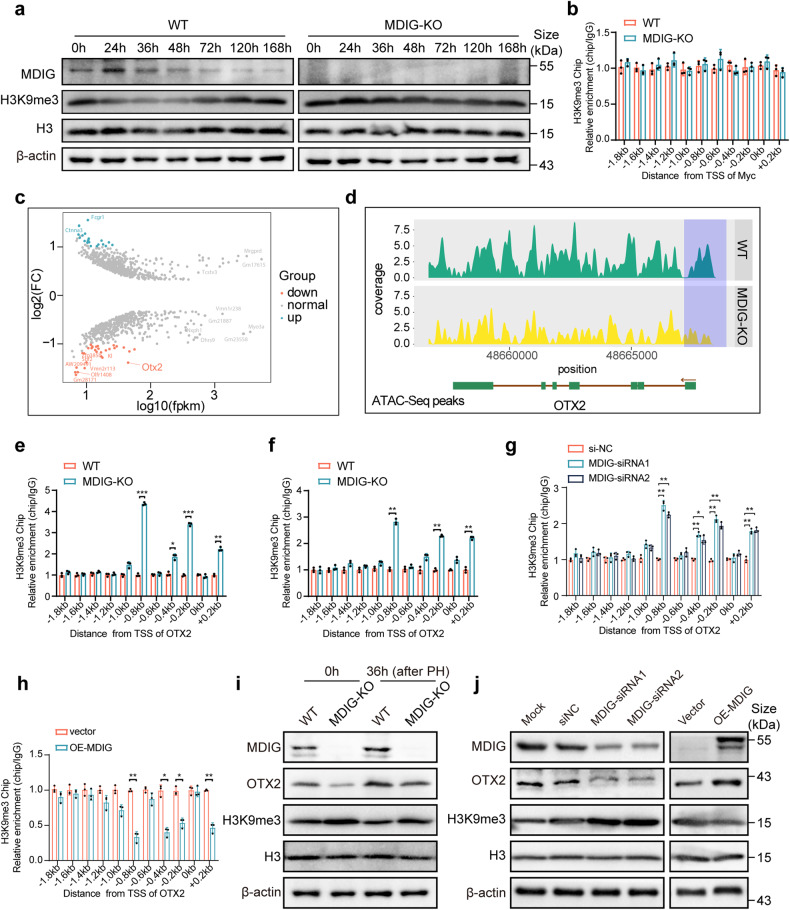
MDIG ablation reduces chromatin accessibility for OTX2 by increasing the H3K9 methylation of its promoter. **a** Western blot for the indicated protein expression in liver tissue lysates prepared from WT and MDIG-KO mice at different times after PH. **b** ChIP-qPCR was conducted to evaluate the enrichment of H3K9me3 in different promoter regions of Myc in liver tissue lysates prepared from WT and MDIG-KO mice at 36 h after PH. **c** Differential ATAC-seq peak analysis between the WT and MDIG-KO mice at 36 h after PH defined 1114 significantly enriched peaks corresponded to 1008 genes. **d** Genome browser view showing ATAC-seq signal around the OTX2 loci. The blue shadow represented the OTX2 promoter-open peak that was correlated with the OTX2 expression. **e**, **f** ChIP-qPCR was conducted to evaluate the enrichment of H3K9me3 in different promoter regions of OTX2 in liver tissue lysates prepared from WT and MDIG-KO mice at 36 h after PH (**e**) and CCl_4_ challenge (**f**). **g**, **h** ChIP-qPCR was conducted to evaluate the enrichment of H3K9me3 in different promoter regions of OTX2 in AML12 cells after silencing (**g**) or overexpressing (**h**) MDIG. **i** Western blot for the indicated protein expression in liver tissue lysates prepared from WT and MDIG-KO mice at 0 h and 36 h after PH. **j** Western blot for indicated protein expression after MDIG knockdown or overexpression in AML12 cells. Data were shown as mean ± SD, unpaired Student’s *t*-test, *P < 0.05; **P < 0.01; ***P < 0.001

As MDIG had been demonstrated to modulate DNA and histone methylation,^15,35^ we next examined H3K9 methylation in regenerating livers after PH. In WT mice, the H3K9me3 was dynamically regulated during liver regeneration, whose levels displayed an opposite change to MDIG. While, the total expression of H3K9me3 was upregulated, with a rapid increase followed by a gradual decrease during liver regeneration after MDIG depletion (Fig. 4a and Supplementary Fig. S10a). To our surprise, knocking out or overexpressing MDIG had no influence on the H3K9me3 at Myc promoter, suggesting that Myc was regulated by MDIG in an indirect manner (Fig. 4b and Supplementary Fig. S10b, c). Since methylation of histones is reported to regulate the chromatin accessibility via forming euchromatin or heterochromatin, we subsequently evaluated the effects of MDIG depletion on chromatin accessibility of livers at 36 h after PH using Assay for Transposase-Accessible Chromatin (ATAC-seq). ATAC-seq results (Accession number: CRA007598) in both WT and MDIG-KO livers revealed open chromatin regions were concentrated around the TSS (Supplementary Fig. S10d). Differential analysis of accessible ATAC-seq peaks identified 1114 peaks that corresponded to 1008 genes, including 561 genes with gained accessibility and 447 with decreased accessibility (Fig. 4c). Loss of MDIG induced upregulation of H3K9me3, which should favor the formation of heterochromatin and reduce chromatin accessibility. Among the genes with decreased accessibility, we only considered those candidates that could uniquely govern Myc expression and showed high expression levels. This highlighted OTX2 as the most highly and uniquely overrepresented gene in MDIG-mediated peaks. During liver regeneration, MDIG deficiency appeared to favor to form the closed chromatin for transcription factor OTX2 (Fig. 4d), without altering the chromatin accessibility of Myc (Supplementary Fig. S10e). In addition, we also performed integrated analysis on the RNA-seq data and the ATAC-seq data. A total of 50 differentially expressed genes presented altered chromatin openness. However, analysis of chromatin accessibility and transcript expression abundance revealed no forward or reverse relationship between these two regulations. In other words, the regulation of these two processes is likely to be independent of each other (Supplementary Fig. S11).

To validate these results, we conducted ChIP-qPCR to examine the distribution of the repressive marker H3K9me3 on the OTX2 promoter region. Eleven pairs of primers were designed to detect possibly altered sites in the OTX2 promoter region. The ChIP-qPCR assay demonstrated a significantly increased enrichment of H3K9me3 at multiple promoter regions of OTX2 in the MDIG-deficient liver at 36 h after PH (Fig. 4e) and CCl_4_ challenge (Fig. 4f). The expression levels of OTX2 mRNA and protein showed initial upregulation followed by downregulation during liver recovery after MDIG depletion (Supplementary Fig. S12a–c). To further confirm this result, we knocked down MDIG in murine hepatocytes cell line AML12 and found the enrichment of H3K9me3 was increased at −1.0 kb, −0.8 kb, −0.4 kb, −0.2 kb, and +0.2 kb promoter regions of OTX2 (Fig. 4g), while the enrichment was decreased after MDIG overexpression (Fig. 4h). Consistent results were also observed in the Hepa1-6 cells (Supplementary Fig. S12d, e). Thus, in the quiescent livers, loss of MDIG induced the upregulation of H3K9me3 and downregulation of OTX2. During the liver regeneration, decreased H3K9me3 appeared to promote the accessibility of OTX2 and thus activated its transcription. However, the protein levels of OTX2 were obviously downregulated in the MDIG-deficient liver at 36 h after PH, with a slight increase of H3K9me3 compared with WT mice (Fig. 4i and Supplementary Fig. S12f). Consistently, OTX2 were dramatically decreased following MDIG-deletion, and increased after MDIG overexpression (Fig. 4j and Supplementary Fig. S12g).

Overall, these findings indicate that alteration of H3K9me3 caused by loss of MDIG appeared to favor the formation of closed chromatin for OTX2.

### OTX2 promotes cell proliferation by enhancing Myc expression

**Fig. 5:**
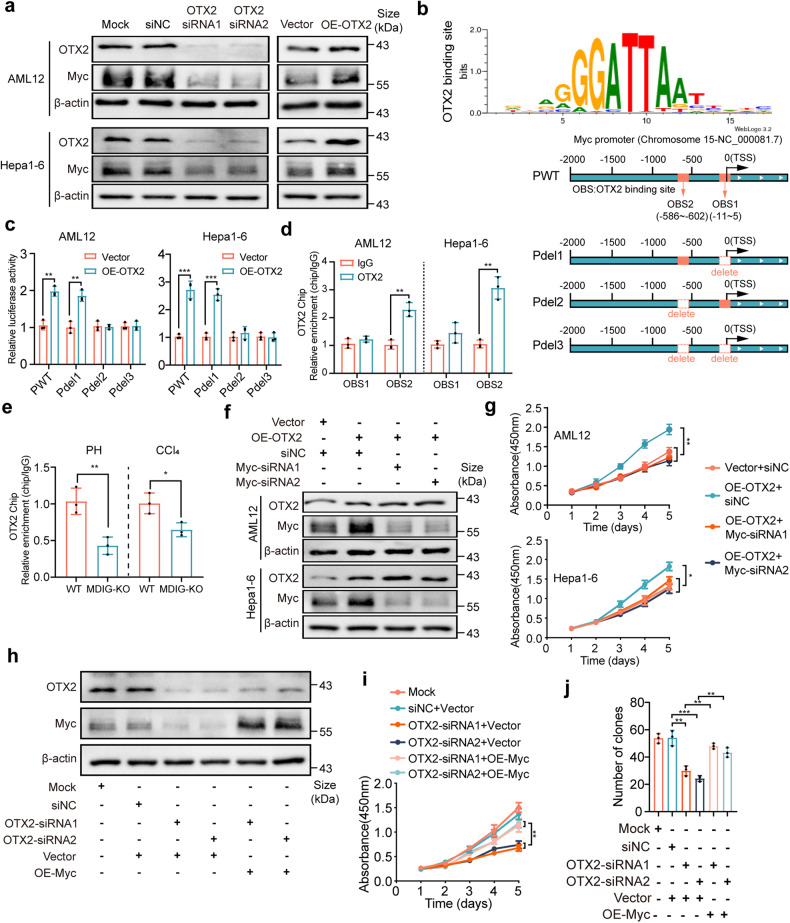
OTX2 promotes cell proliferation by enhancing Myc expression. **a** Western blot analysis showing the expression of OTX2 and Myc after silencing or overexpressing OTX2 in AML12 and Hepa1-6 cells. **b** Top, the schematic indicated the sequence logo of OTX2 potential binding site in JASPAR software. Bottom, wildtype (wt) and deleted mutation (del) recognition sites of OTX2 in the Myc promoter region; PWT, wild-type OTX2 recognition site; Pdel1, deleted OTX2 recognition site 1; Pdel2, deleted OTX2 recognition site 2; Pdel3, deleted OTX2 recognition site 1 and site 2. **c** The relative activities of the Myc promoter and the deleted promoter after transfection of OTX2 and Vector. **d** ChIP-qPCR assays with anti-OTX2 or negative control (anti-IgG) antibodies showed OTX2 binding to the recognition site 2 of Myc promoter in AML12 and Hepa1-6 cells. ChIP enrichments were normalized to the input signal. **e** ChIP-qPCR assays to analyze OTX2 occupancy at Myc promoter in liver tissue lysates prepared from WT and MDIG-KO mice at 36 h after PH or CCl_4_ challenge. **f** Western blot analysis showing the expression of OTX2 and Myc after silencing Myc in AML12 and Hepa1-6 cells overexpressing OTX2. **g** CCK8 assays revealed cell proliferation capacity after silencing Myc in OTX2-overexpressing AML12 and Hepa1-6 cells. **h** Colony formation assays (left) and EdU immunofluorescence assays (right) revealed cell proliferation capacity after silencing Myc in OTX2-overexpressing Hepa1-6 cells. **i** Western blot analysis showing the expression of OTX2 and Myc after overexpressing Myc in OTX2-silencing Hepa1-6 cells. **j** CCK8 assays revealed cell proliferation capacity after overexpressing Myc in OTX2-silencing Hepa1-6 cells. Data were shown as mean ± SD, unpaired Student’s *t*-test, *P < 0.05; **P < 0.01; ***P < 0.001

OTX2 has been recently implicated in regulating the cell cycle and proliferation of tumors.^36,37^ Our data demonstrated that overexpression of OTX2 promoted cell proliferation, while silencing of OTX2 inhibited cell proliferation as revealed by CCK8, colony formation, and EdU assays (Supplementary Fig. S13a–c). Next, we determined whether OTX2 could regulate Myc expression. As expected, silencing of OTX2 in AML12 and Hepa1-6 cells inhibited Myc expression, while overexpression of OTX2 induced Myc upregulation (Fig. 5a). We further investigated the mechanism underlying OTX2 regulated Myc expression. Transcription factors are implicated in transcription by recognizing and binding to sequences motif located at the promoter. Thus, we first analyzed the possible binding site of OTX2 at the Myc promoter region. JASPAR database identified two OTX2 binding sites (OBS) in the promoter region of Myc (Fig. 5b, top). To determine the transcriptional activation of OTX2 at the Myc promoter, dual-luciferase assays were conducted using wildtype or deleted OBS sequences that were cloned to tandem Renilla and Firefly luciferase reporters (Fig. 5b, bottom). OTX2 overexpression markedly enhanced the luciferase activity of the Myc promoter, and deletion of OBS2, rather than other sites, weakened OTX2-mediated promoter reporter activity of Myc (Fig. 5c). In addition, ChIP-qPCR assays confirmed the high enrichment of OTX2 on OBS2 at the Myc promoter (Fig. 5d). Moreover, OTX2 binding at the promotor region of the Myc gene was decreased in MDIG-KO mice compared to WT mice at 36 h after PH and CCl_4_ challenge (Fig. 5e), supporting that OTX2 directly downregulated Myc expression in MDIG-deficient livers. Meanwhile, the functional experiments demonstrated that Myc knockdown attenuated cell proliferation that were enhanced by OTX2 overexpression (Fig. 5f, g and Supplementary Fig. S13d, e). Conversely, the reintroduction of Myc restored the cell proliferation induced by OTX2 silencing (Fig. 5h–j and Supplementary Fig. S13f, g).

Collectively, these data indicated that OTX2 facilitated cell proliferation by enhancing Myc expression.

### MDIG promotes Myc expression by upregulation of OTX2

**Fig. 6:**
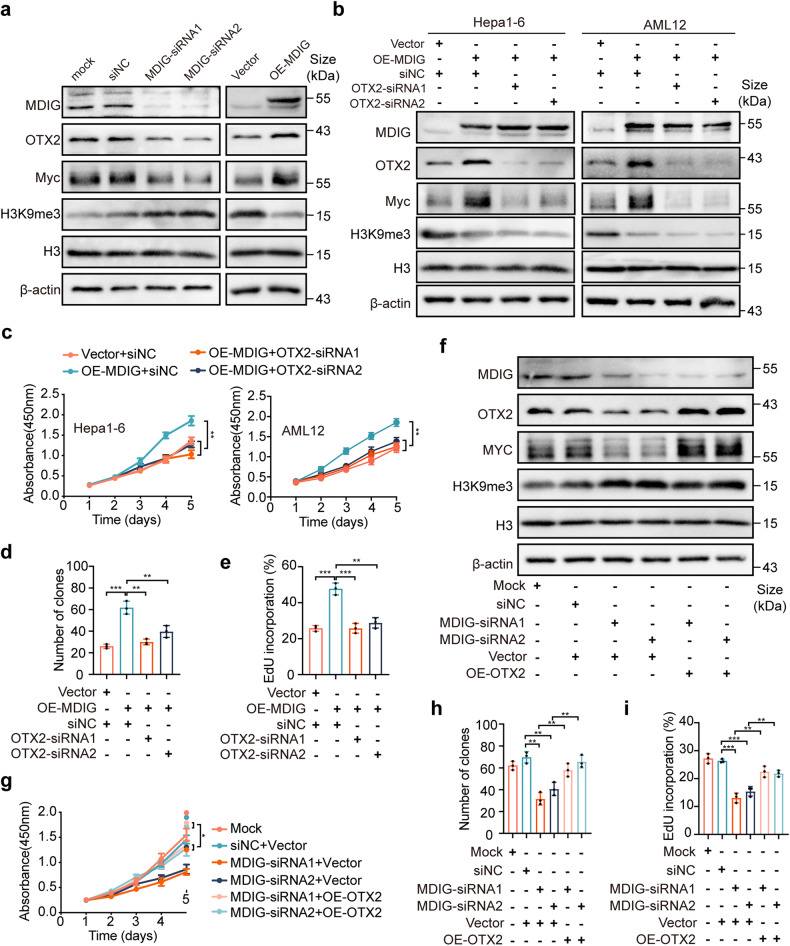
MDIG promotes Myc expression by upregulation of OTX2. **a** Western blot for the indicated protein expression after MDIG knockdown or overexpression in Hepa1-6 cells. **b** Western blot analysis for the indicated protein expression after silencing OTX2 in MDIG-overexpressing AML12 and Hepa1-6 cells. **C** CCK8 assays revealed cell proliferation capacity after silencing OTX2 in MDIG-overexpressing AML12 and Hepa1-6 cells. **d** Colony formation assays and **e** EdU immunofluorescence assays revealed cell proliferation capacity after silencing OTX2 in MDIG-overexpressing Hepa1-6 cells. **f** Western blot for the indicated protein expression after overexpressing OTX2 in MDIG-silencing Hepa1-6 cells. **g** CCK8 assays revealed cell proliferation capacity after overexpressing OTX2 in MDIG-silencing Hepa1-6 cells. **h** Colony formation assays revealed colony formation capacity after overexpressing OTX2 in MDIG-silencing Hepa1-6 cells. **i** EdU immunofluorescence assays revealed cell proliferation capacity after overexpressing OTX2 in MDIG-silencing Hepa1-6 cells. Data were shown as mean ± SD, unpaired Student’s *t*-test, *P < 0.05; **P < 0.01; ***P < 0.001

To further investigate whether MDIG promotes Myc expression by upregulation of OTX2, a western blot was conducted to detect the expression of OTX2, Myc, and H3K9me3 after modulating MDIG. Knocking down of MDIG induced a noticeable downregulation of OTX2 and Myc and upregulation of H3K9me3 levels. However, overexpression of MDIG resulted in an opposite effect (Fig. 6a). Similar results were also observed in MDIG-deficient livers after PH. In quiescent livers, we detected the upregulation of H3K9me3 in MDIG-KO livers, with a slight decrease in OTX2 and Myc expression. The PH exaggerated the variation of these protein expressions of both groups (Supplementary Fig. S14a). We next explored the role of OTX2 in the upregulation of Myc caused by MDIG. As expected, silencing of OTX2 could attenuate the upregulation of Myc induced by MDIG overexpression, which also reduced the levels of H3K9me3 (Fig. 6b). Functionally, CCK8, colony formation, and EdU assays showed that MDIG overexpression promoted cell proliferation, which could be partially abrogated by OTX2 knockdown (Fig. 6c–e and Supplementary Fig. S14b). On the other hand, the knockdown of MDIG increased the levels of H3K9me3 and decreased the levels of Myc, which could be restored by OTX2 overexpression (Fig. 6f). Meanwhile, the suppression of cell proliferation and clonogenicity caused by MDIG silencing was rescued by OTX2 upregulation (Fig. 6g–i and Supplementary Fig. S14c–e). The results suggest that OTX2 facilitated cell proliferation by enhancing Myc expression.

Next, we questioned whether OTX2 overexpression could restore the effects caused by the loss of MDIG in vivo. For this purpose, we introduced AAV-OTX2, with AAV-EGFP as the control, in the MDIG-KO mice and established a CCl_4_ liver injury model. To our excitement, overexpression of OTX2 significantly promoted liver recovery as measured by necrotic scores as well as levels of liver enzymes. In addition, IHC analysis of the cell proliferation markers suggested the robust proliferating cells in the OTX2-MDIG-KO group, further confirming that OTX2 rescued the phenotypes caused by the loss of MDIG (Supplementary Fig. S15).

In summary, these findings supported that MDIG promoted Myc expression by upregulation of OTX2.

### MDIG contributes to liver regeneration through the Myc-MDIG-positive feedback loop

**Fig. 7:**
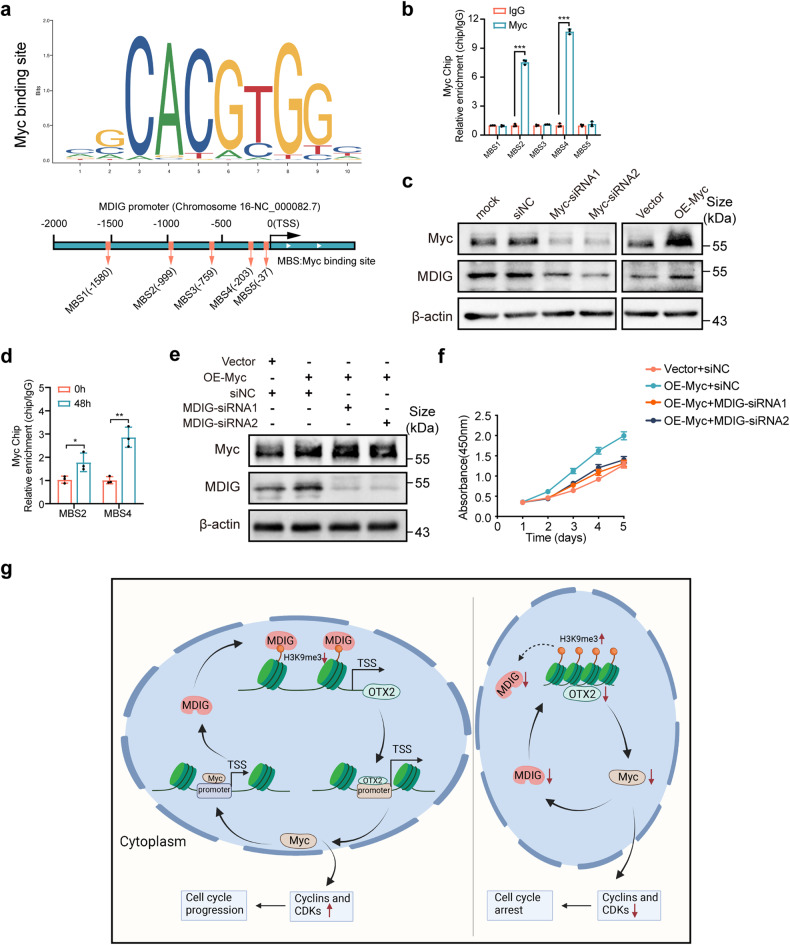
MDIG contributes to liver regeneration through the Myc-MDIG-positive feedback loop. **a** Top, the schematic indicated the sequence logo of Myc potential binding site in JASPAR software. Bottom, schematic outlined of the putative binding of Myc to MDIG gene promoter region. **b** ChIP-qPCR assays with anti-Myc or negative control (anti-IgG) antibodies showed Myc binding to the recognition sites 2 and 4 of MDIG promoter in Hepa1-6 cells. ChIP enrichments were normalized to the input signal. **c** Western blot analysis showing the expression of Myc and MDIG after silencing or overexpressing Myc in Hepa1-6 cells. **d** ChIP-qPCR assays with an anti-Myc or negative control (anti-IgG) antibodies showed increased occupancy of Myc at the recognition sites 2 and 4 of MDIG promoter at 48 h after PH. ChIP enrichments were normalized to the signal from 0 h samples. **e** Western blot for the indicated protein expression after overexpressing Myc in MDIG-silencing AML12 cells. **f** CCK8 assays revealed cell proliferation capacity after overexpressing Myc in MDIG-silencing AML12 cells. **g** A proposed model illustrating promoted effects of MDIG on liver regeneration (This graphic figure was created in BioRender.com). Data were shown as mean ± SD, unpaired Student’s *t*-test, ***P < 0.001

Given that MDIG upregulation after PH was important to induce Myc expression in the regenerating livers and sustained regenerative signaling, we wanted to identify the upstream signal that regulated MDIG expression. MDIG, also known as Myc-induced nuclear antigen (MINA), was first confirmed as a nuclear protein transcriptionally modulated by Myc in humans,^19^ indicating that Myc might upregulate MDIG to form a positive feedback loop during liver regeneration. Based on the prediction from JASPAR, we found five potential binding sites of Myc (MBS) in MDIG promoter region (Fig. 7a). To confirm this, we performed ChIP-qPCR assays and found that endogenous Myc was recruited to MBS2 and MBS4 of the MDIG promoter (Fig. 7b). In agreement with the findings, western blot results showed that Myc indeed promoted MDIG expression (Fig. 7c). Moreover, the ChIP-qPCR assays also confirmed the occupancy of Myc at MDIG promoter region significantly increased in the regenerating liver cells (48 h after injury) compared to the quiescent liver cells (0 h after injury; Fig. 7d). Interestingly, we also noted a slight increase in the expression of MDIG at the very late stage of liver recovery in the CCl_4_ model (Supplementary Fig. S12c). In addition, overexpression of Myc could rescue the growth inhibition effect caused by silencing MDIG in vitro (Fig. 7e, f).

In summary, the results suggest that MIDG induced upregulation of Myc at the initial stage could, in turn, promotes MDIG transcribe as Myc facilitates its transcription, constituting a positive feedback loop that sustains liver regeneration (Fig. 7g).

## Discussion

The adult livers exhibit an efficient regenerative ability in response to liver injury, during which plenty of pro-regenerative genes are activated to drive the compensatory proliferation of the remanent hepatocytes.^1,6^ Epigenetic modifications are implicated in liver regeneration with studies describing histone acetylation, m6A modification, and DNA methylation.^29,38,39^ However, the role of histone methylation on liver regeneration is still unsolved. In the present study, we revealed the dynamic alterations of histone methylation during liver regeneration. Mechanistically, MDIG, a histone demethylase towards H3K9me3, was identified here to be critical for regulating histone methylation by mediating chromatin accessibility of genes associated with the cell cycle. Hepatic ablation of MDIG impaired liver regeneration and increased total H3K9me3 after PH or CCl_4_ challenge, suggesting that MDIG is a pivotal regulator during liver regeneration. Of note, MDIG somatic mutation could be found in ~2% of human cancer patients, most of which are missense or synonymous mutation^40,41^ (Supplementary Fig. S16). However, such mutations need to be further investigated to determine whether it is a gain of function mutation or loss of function mutation. Also, as MDIG functions as a target downstream of Myc and drugs targeting Myc are still less effective, selecting small molecules that could modulate MDIG might be a promising option for the treatment of Myc-related disease.

Liver regeneration following injury is accompanied by multiple alterations in gene expression, which is precisely regulated by the coordination of transcriptional regulators and complex epigenetic modifications, including histone posttranslational modifications, chromatin remodelers, DNA modifications, and other molecular events.^9,23,42^ The compensatory action between DNA methylation and histone methylation demonstrated by Wang et al. might explain how pro-regenerative genes are remodeled by epigenetic code during liver regeneration. The loss of DNA methylation of transposons caused by UHRF1 deletion in hepatocytes induces H3K27me3 redistribution from pro-regeneration genes to hypomethylated transposons to silence transposon, resulting in enhanced liver regeneration.^39^ Many previous studies have uncovered the crucial role of regional modifications of histone tails in regulating chromatin dynamics, and aberration of these processes usually induces tumorigenesis. The methylation status on histone has been shown to have of close association with transcriptional regulation and can either activate or suppress gene transcription.^12,43^ Generally, the lysine methylations on H3K9, H3K27, and H4K20 tend to inhibit gene transcription,^44^ and the most common function of H3K9me3 is to maintain the heterochromatin, an architecture related to repressed transcription. Conversely, demethylating of tri- to mono-methyl from H3K9 frequently promotes gene transcription.

Our results identified the dynamic changes in H3K9me3 modifications during liver regeneration. In addition, hepatic ablation of MDIG led to increased expression of H3K9me3, which underwent initial upregulation and subsequently downregulation during liver regeneration, supporting that MDIG-mediated demethylation of H3K9me3 played a key role in regulating liver regeneration. In fact, whole genome analysis of H3K9me3 before and after MDIG deletion by using the Cleavage Under Targets and Tagmentation (CUT&Tag) assays further confirmed this hypothesis. MDIG-KO led to almost equivalent reads abundance at the promoter region (−2.0 kb to TSS), but it changed the modes of interactions between H3K9me3 and transcriptional factors (TFs). Specifically, MDIG-KO favors the binding of H3K9me3 on the promoter region (−1.0 kb to TSS) and the Exons. Interestingly, MDIG deletion affects the interaction between H3K9me3 and OTX2, rather than MYC (or MYC-MAX). Intriguingly, MDIG-dependent modifications of H3K9me3 were also found among TFs that govern liver cell differentiation (HNF4A, HES1, and SOX14), and oxidative stress (HSF1, HSF2, NFE2L2, and HIF1A). Of note, silencing of MDIG also resulted in increased levels of cell cycle repressors P21 and P27, and vice versa (Supplementary Fig. S17). Taken together, the loss of MDIG-induced upregulation of H3K9me3 at promoters of multiple TFs and cell cycle regulators, primed for inactivation in the quiescent liver, created an unfavorable epigenetic environment for subsequent liver regeneration and repressed hepatocytes proliferation.

Changes in chromatin accessibility can regulate gene expression by altering the binding ability of transcription factors to target DNA recognition sequences.^43^ The chromatin gets repatterned in regenerating livers so that pro-proliferation genes shift to more accessible to transcriptional factors assembled on regions of open chromatin, enabling the hepatocytes entry into the cell cycle.^45^ A recent ATAC-seq profiling uncovered that Arid1a in the SWI/SNF complex promoted liver regeneration by setting a permissive chromatin state in hepatocytes, allowing for rapid activation of liver-progenitor-like cell genes.^46^ Our results showed that such permissive regions reduced in MDIG-KO hepatocytes, as demonstrated by a group of genes with decreased accessibility during liver regeneration. However, no changes in chromatin accessibility of Myc warranted our further investigation to understand its contribution to mediating MDIG-induced liver regeneration. OTX2, a TF that was reported to regulate Myc expression, showed a significant decrease in chromatin accessibility at its promoter. Repression of chromatin accessibility might then cause inactivation of the respective genes by OTX2 for which chromatin accessibility and expression were also decreased upon MDIG depletion in hepatocytes. Interestingly, the reduced enrichment of OTX2 at the Myc promoter region in MDIG-KO hepatocytes indicated an MDIG- and OTX2-dependent inactivation of Myc during liver regeneration. Therefore, it was possible that the preexisting chromatin status of individual genes might affect chromatin accessibility induced by MDIG. When the chromatin of the OTX2 promoter was in a partial opening status in quiescent hepatocytes, loss of MDIG elevated the levels of H3K9me3, inducing the formation of heterochromatin and repressing the expression of OTX2. The most characteristic of heterochromatin is marked by DNA methylation, repressive histone modifications such as H3K9me3 or H3K27me3 that allow the chromatin to remain inaccessible to regulatory factors.^47,48^ The significantly increased enrichment of H3K9me3 at promoter regions of OTX2, rather than Myc, in the MDIG-deficient liver explained the difference in chromatin accessibility between OTX2 and Myc. However, the mechanisms of improved H3K9me3 at certain groups of genes (such as OTX2, not Myc) in response to MDIG deletion during liver regeneration were not fully understood. Whether some molecules mediated the recruitment of MDIG to the OTX2 promoter would warrant further studies. Of note, the primary hepatocytes could be more representative, than the AML12 cell lines, as the tool for mechanistic studies. Unfortunately, we found that the cells isolated from the MDIG-KO liver grew very slowly and eventually underwent cell death. It is likely that the primary hepatocytes critically rely on MDIG’s promoting effects on cell proliferation.

Myc is generally considered to function as a transcriptional activator that coordinates multiple genes to control somatic cell proliferation, as well as tissue regeneration. Genome-wide profiling of transcriptional responses to Myc reveals that Myc can modulate a great number of genes expression.^49,50^ The activation of ectopic Myc induces a robust transcriptional and proliferative response in the liver. Comprehensive analysis of Myc transcriptional output indicates that Myc binds to a set of target genes at open chromatin involved in regulating cell cycle progression transcriptionally.^51^ In this study, MDIG deletion repressed Myc expression, which slowed down the cell cycle progression. Interestingly, silencing of MDIG induced no changes in the enrichment of H3K9me3 at the promoter regions of Myc, as well as chromatin accessibility of the Myc gene, indicating that the demethylation of MDIG was target-specific and the MDIG-deficient hepatocytes retained an open chromatin state at Myc gene. Therefore, if Myc could be induced in response to regenerative stimulus in the liver, hepatic epigenome architecture did not preclude liver regenerative capacity: rather, it was the MDIG-mediated repressive expression of OTX2 required to drive Myc transcription that thwarted liver regeneration. In addition, Myc also bound to promoter regions of MDIG to potentiate its transcription initiation, forming a positive feedback loop to sustain hepatocytes proliferation during regeneration. However, as such feedback depends on the binding of Myc and MDIG promoter region while Myc expression peaked at 48 h after damage (Supplementary Fig. S12b, c), we reasoned that Myc might not be the main regulator of MDIG at the early time points of liver recovery.

One important question to be solved is whether the expression changes of MDIG exhibit cell heterogeneity during liver regeneration. We analyzed the expression of MDIG at different time points after damage in two main sources of regenerative liver cells (hepatocytes and cholangiocytes) using immunofluorescence co-staining assays. The results showed that both hepatocytes and cholangiocytes expressed MDIG during liver regeneration. Interestingly, we found that MDIG underwent an expansion from the peri-central region to the peri-portal region during the recovery. (Supplementary Fig. S18). However, as several other cell types (hepatic stellate cells, Kupffer cells, and liver sinusoidal endothelial cells, *etc*.) also involve in liver recovery, high throughput sequence on the single cell scale using samples from different regenerating stages in combination with lineage-tracing strategy might provide more precise answers to this question. Such findings could also be investigated and validated with the help of genetically engineered lineage-tracing mice.

In conclusion, our data suggest that liver regenerative capacity is tightly linked to hepatic chromatin states. The H3K9me3 demethylase MDIG plays an essential role in promoting liver regeneration, at least partly via enhancing the chromatin accessibility of the OTX2 promoter region, which induces Myc expression and thereby drives hepatic regenerative biological output.

## Materials and methods

### Animals

The experiments were approved by the Animal Ethic Review Committees of the West China Hospital, China. All MDIG wild-type (WT) and liver-specific MDIG knockout (MDIG-KO) mice on a *C57BL/6J* background were generated from MDIG heterozygous mice. *MDIG*^*loxp/loxp*^ mice with a loxP-flanked MDIG allele and Albumin-Cre mice were obtained from Gempharmatech Co., Ltd (Nanjing, China). The *MDIG*^*loxp/loxp*^ mice were bred with Albumin-Cre mice to generate MDIG-KO mice. The tail DNAs were used to confirm the genotypes by PCR amplification. An equal number of male and female mice in each group were used in this study to exclude the influences from gender. For 70% PH surgery, the mice were removed 70% of liver mass as previously described.^30^ For the acute liver injury model, the mice were challenged by intraperitoneal injection of 10 ml/kg body weight of CCl_4_ (10% CCl_4_ in corn oil) as previously described.^26^ The mice were intraperitoneally injected with 1 mg/kg body weight BrdU one hour before sacrifice. (cat. B5002; Sigma). For overexpressing of EGFP, MDIG, OTX2 in the mouse liver, AAV-EGFP, AAV-MDIG, and AAV-OTX2 were diluted in the PBS to 100ul (6 × 10^11^ vg/ml) and delivered to the mouse liver through the tail vein, respectively, 4 weeks before PH or CCl_4_ treatment.

### Generation of *MDIG*^*loxp/loxp*^ mouse model

Clustered, regularly-interspaced short palindromic repeats (CRISPR)/Cas9 technology was used to generate *MDIG*^*loxp/loxp*^ mouse model based on a *C57BL/6* *J* background. Briefly, 2 guide RNAs (gRNAs) targeting MDIG introns 2 and 6, respectively, were used to construct the MDIG-floxed strain, in which exons 3–5 of the MDIG allele were flanked by loxp sites. The sequence of gRNA targeting intron 2 was 5’-ATCGCTCTGATTTTGATGGC-3’. The sequence of gRNA targeting intron 6 was 5’- CGTACTTAGTCACTCTGATG-3’. The MDIG donor vector containing loxp sites flanking exons 3–5 and 2 homology arms, together with Cas9 mRNA, was introduced into *C57BL/6J* fertilized eggs. The positive founder mice were crossed with WT C57BL/6J mice to obtain MDIG loxp homozygous mice (Supplementary Fig. S1d). Progeny were screened by PCR for germ-line transmission of the targeted alleles. The PCR products were further confirmed by sequencing. MDIG^loxp/loxp^ homozygous mice were mated to Albumin-Cre mice to generate MDIG^loxp/loxp^/Albumin-Cre mice.

### Immunohistochemistry (IHC) and immunofluorescence (IF)

Paraformaldehyde solution was used to fix liver specimens for 24 h. Paraffin sections (4 μm thick) were deparaffinized and rehydrated, followed by antigen retrieval, and then the tissue sections were incubated with the corresponding primary antibodies at 4 °C overnight. The chromogenic reaction was performed using a DAB solution for IHC assays. Primary antibodies were BrdU (cat. 5292; CST), pH3S10 (cat. 06-570; Merck Millipore), and Ki67 (cat. ab16667; Abcam). For IF, primary antibodies were CK19 (cat. ab52625, Abcam), ALB (cat. 16475-1-AP, Proteintech), MDIG (cat. 12214-1-AP; Proteintech), Donkey anti-Rabbit IgG (H + L) Highly Cross-Adsorbed Secondary Antibody Alexa Fluor™ Plus 555 (cat. A32794, Thermo Fisher), iFluor^®^ 430 Tyramide (cat. 45096, AATbio), iFluor^®^ 488 Tyramide (cat. 11060, AATbio) were used. Visualization of immunofluorescence was observed with a confocal laser scanning microscope (Leica, DM-8).

### Western blot analysis

Protein extraction was conducted using RIPA Lysis Buffer (Beyotime Biotechnology, Shanghai, China) containing a protease inhibitor cocktail (Thermo Fisher Scientific, USA). Sodium dodecyl sulfate-polyacrylamide gel electrophoresis (SDS-PAGE) assay was performed as in previous reports.^52^ Primary antibodies used in this study were β-actin (cat. 200068-8F10; ZEN-bioscience), H3 (cat. ASO71; ABclonal), Cyclin D1 (cat. 55506 T; CST), CDK4 (cat. 12790 T; CST), Cyclin A2 (cat. ab181591; Abcam), Cyclin E1 (cat. 20808 S; CST), CDK1 (cat. ab32094; Abcam), Cyclin B1(cat. 55004-1-AP; Proteintech), H3K9me1 (cat. A2358; ABclonal), H3K9me2 (cat. A2359; ABclonal), H3K9me3 (cat. ab176916; Abcam), MDIG (cat. 12214-1-AP; Proteintech), OTX2 (cat. 13497-1-AP; Proteintech), Myc (cat. 10828-1-AP; Proteintech), BRCA2 (cat. AF7817; Affinity), Junb (cat. 10486-1AP; Proteintech), E2F2 (cat. AF4100), ATAD5 (cat. Bs-7656R; Bioss). Western blots were quantified using ImageJ software (Version 1.51n, National Institutes of Health, Bethesda, MD, https://imagej.net/ij/index.html), as previously described.^53^ The original and uncropped films of Western blots were provided as Supplementary Figs. S19–S21.

### Cell culture, Small interfering RNA transfection, plasmid transfection, and lentiviral infection

The mouse hepatoma cell line Hepa1-6, cultured in Dulbecco’s modified Eagle’s medium (DMEM) containing 10% fetal bovine serum, was maintained at 37 °C in a humidified atmosphere containing 5% CO_2_. Small interfering RNA (siRNA) was purchased from RiboBio Co., Ltd. (Guangzhou, China), which was transfected using Genmute^TM^ Reagent (SignaGen Laboratories, Maryland, USA). The target sequences of siRNAs were listed in Supplementary Table S4. GenJet^TM^ plus Reagent (SignaGen Laboratories, Maryland, USA) was used for plasmid transfection. The *MDIG* gene was cloned into a lentiviral package vector to overexpress MDIG, which was synthesized by GENECHEM (Shanghai, China). Stable infection was performed according to standard procedures.

### Cell Counting Kit-8 (CCK8) assay, cell cycle analysis, colony formation assays, and EdU (5-Ethynyl-2’-deoxyuridine) labeling assay

CCK8 assay (Beyotime Biotechnology, Shanghai, China) was conducted to determine cell viability. In brief, 100 μl of culture media containing 2.0 × 10^3^ cells was added into 96-well plates. At indicated time point, 10 μL of CCK8 solution was added into each well and the absorbance was measured at 450 nm by the Eon^TM^ Microplate Reader (BioTek, VT, USA). Cell Cycle Kit (4ABiotech, Beijing, China) was used to determine the cell cycle following the manufacturer’s instructions. After labeling with PI, cells were examined using CytoFLEX Research Flow Cytometer (Beckman Coulter, CA, USA). For colony formation assays, 1.0 × 10^3^ cells were seeded in each well of a 12-well plate. Two weeks later, cells were fixed by 4% paraformaldehyde, followed by staining with 0.1% crystal violet. The results were analyzed using ImageJ software (National Institutes of Health, Bethesda, MD, USA). For the EdU labeling assay, cells were planted into a 24-well plate at a density of 2.0 × 10^4^ in triplicate. Cell-Light^TM^ Apollo 567 Stain Kit (RiboBio Biotechnology, Guangzhou, China) was used to assess cell proliferation according to the manufacturer’s instructions. After staining, cells were photographed using OBSERVER D1/AX10 cam HRC microscope (Zeiss, Oberkochen, Germany).

### ATAC-seq

The ATAC-seq program was performed at SeqHealth Tech Co., Ltd (Wuhan, China). The liver tissue was homogenized into cell suspension. About 5 × 10^4^ cells were used to prepare nuclei. After centrifugation, the cell pellet was resuspended with 50 μl chilled lysis buffer, followed by centrifuging at 500 × *g* for 10 min. After removing the supernatant, the nuclei pellet was resuspended using the transposase reaction mix and purified using a MinElute PCR Purification kit (Cat. 28006; Qiagen). Following purification, library fragments was amplificated in 1 × Nextera PCR master mix (Cat. M0541S; NEB) and Nextera PCR primers 1 and 2. Following amplification, a Qiagen MinElute Purification kit was used to yield a final library concentration of ~30 nM in 20 μL. Subsequently, libraries were sequenced on the Hiseq 3000 platform for pair-end 50 bp sequencing.

### RNA sequencing

The mRNA sequencing program was performed at LC-Bio Technology Co., Ltd (Hangzhou, China). Briefly, the total RNAs were isolated from liver samples using TRIzol agent (Invitrogen, CA, USA). After establishing sequencing libraries, samples were subjected to perform the 2 × 150 bp paired-end sequencing (PE150) on an Illumina Novaseq™ 6000.

### CUT&Tag sequence

CUT&Tag assay was performed using HyperactiveTM In-Situ ChIP Library Prep Kit for Illumina (Cat. TD901-TD902, Vazyme Biotech, China) according to the manufacturer’s instruction. Library preparation was performed by OE Biotech Co., Ltd (Shanghai, China) and H3K9me3 primary antibody (Cat. 39161, Thermo Fisher Scientific, Waltham, MA) was used. Libraries were sequenced on the Illumina NovaSeq6000 platform and 150 bp paired-end reads were generated for the following analysis. Fastp software and Bowtie2 were used for genome alignment (Mus musculus, mm10, GRCm38.p6)). Then, the SEACR software was applied to identify genomic regions with multiple overlapping DNA peaks enrichment for the prediction of putative binding sites. Visualization of peak distribution along genomic regions of interested genes was performed with IGV. Peaks were then annotated using chipseeker software to obtain the genes and gene annotations about peaks. Significant motifs of peaks were analyzed by MEME and DREME software and aligned to the motif database. Different peaks between case and control groups were analyzed using Manorm software.

### Quantitative reverse transcription PCR (qRT-PCR)

Total RNA isolation was performed using Cell Total RNA Isolation Kit (Foregene Biotech, Chengdu, China). 1 μg total RNA was reverse transcribed to cDNA by HiScript II Q RT SuperMixfor qPCR (+gDNA wiper) (Vazyme Biotech, Nanjing, China). Next, ChamQ™ SYBR@ qPCR Master Mix (Vazyme Biotech, Nanjing, China) was used for real-time PCR. The gene expression was normalized by β-actin and quantitated by the 2^−ΔΔ^ Ct method.

### ChIP-qPCR

ChIP was performed using SimpleChIP Enzymatic Chromatin IP Kit (CST, 9003). Briefly, cells were crossed link with 1% formaldehyde for 10 min, followed by incubating with glycine (0.2 g/mL) for 10 min to stop the reaction. Micrococcal Nuclease added into lysis buffer was used to digest DNA into approximately 1–2 nucleosomes. After that, 5 ug of specific antibody or normal IgG was added into tubes containing cells lysates and incubated at 4 °C overnight, after which protein G magnetic beads were added incubating at 4 °C for 120 min so that the DNA-protein-antibody complexes could be immobilized on the beads. After the beads were washed, DNA fragments were purified, which was then subjected to qRT-PCR to quantify the enrichment. Primers used to amplify the promoter regions of OTX2 and MDIG were listed in Supplementary Table S5.

### Dual-luciferase reporter assay

The normal and mutant promoter region of Myc was synthesized and subcloned into the pEZX-PL01 vector (GeneCopoeia, CA, USA). The Hepa1-6 cells were transiently transfected with the pEZX-PL01-Myc plasmid together with OTX2 or Vector using GenJet^TM^ plus Reagent (SignaGen Laboratories, Maryland, USA). After 48 h, cells were harvested and determined by Dual-Luciferase Reporter Assay Kit (DL101-01, Vazyme) according to the manufacturer’s instructions.

## Statistical analysis

The data were expressed as mean ± standard deviation (S.D). A two-sided Student’s *t*-test was used for the comparisons of continuous variables. Values of *P* < 0.05 were considered statistically significant (*P < 0.05; **P < 0.01; ***P < 0.001). The statistical analysis was performed by Prism version 7.0 software (GraphPad Software).

### Supplementary information

Supplementary Materials

Supplementary Tabe S1

Supplementary Tabe S2

Supplementary Tabe S3

Supplementary Tabe S4

Supplementary Tabe S5

## Data Availability

The raw data of RNA-seq, ATAC-seq, and CUT&Tag assays were deposited in the Genome Sequence Archive (GSA) (https://ngdc.cncb.ac.cn/gsub/), under accession numbers CRA007599, CRA007598, and CRA011318. The other data that support the findings of this study are available from the corresponding author upon reasonable request.
