# Supplementary material for: MDIG-mediated H3K9me3 demethylation upregulates Myc by activating OTX2 and facilitates liver regeneration
Source: Signal Transduct Target Ther. 2023 Sep 15;8:351. doi: 10.1038/s41392-023-01575-5 (PMC10502063; doi:10.1038/s41392-023-01575-5)
Supplement: Supplementary file 1 — Supplementary Materials [file 41392_2023_1575_MOESM1_ESM.docx]

Supplementary Materials for

**MDIG-Mediated H3K9me3 demethylation Upregulates Myc by Activating OTX2 and Facilitates Liver Regeneration**

Jinpeng Du, Wenwei Liao, Haichuan Wang,Guimin Hou, Min Liao, Lin Xu, Jiwei Huang, Kefei Yuan, Xiangzheng Chen, Yong Zeng

Correspondence to:

Xiangzheng Chen (cxz090709@163.com); Yong Zeng (zengyong@medmail.com.cn).

**This PDF file includes:**

Supplementary Figures S1 to S21

Supplementary Tables S1 to S5

**
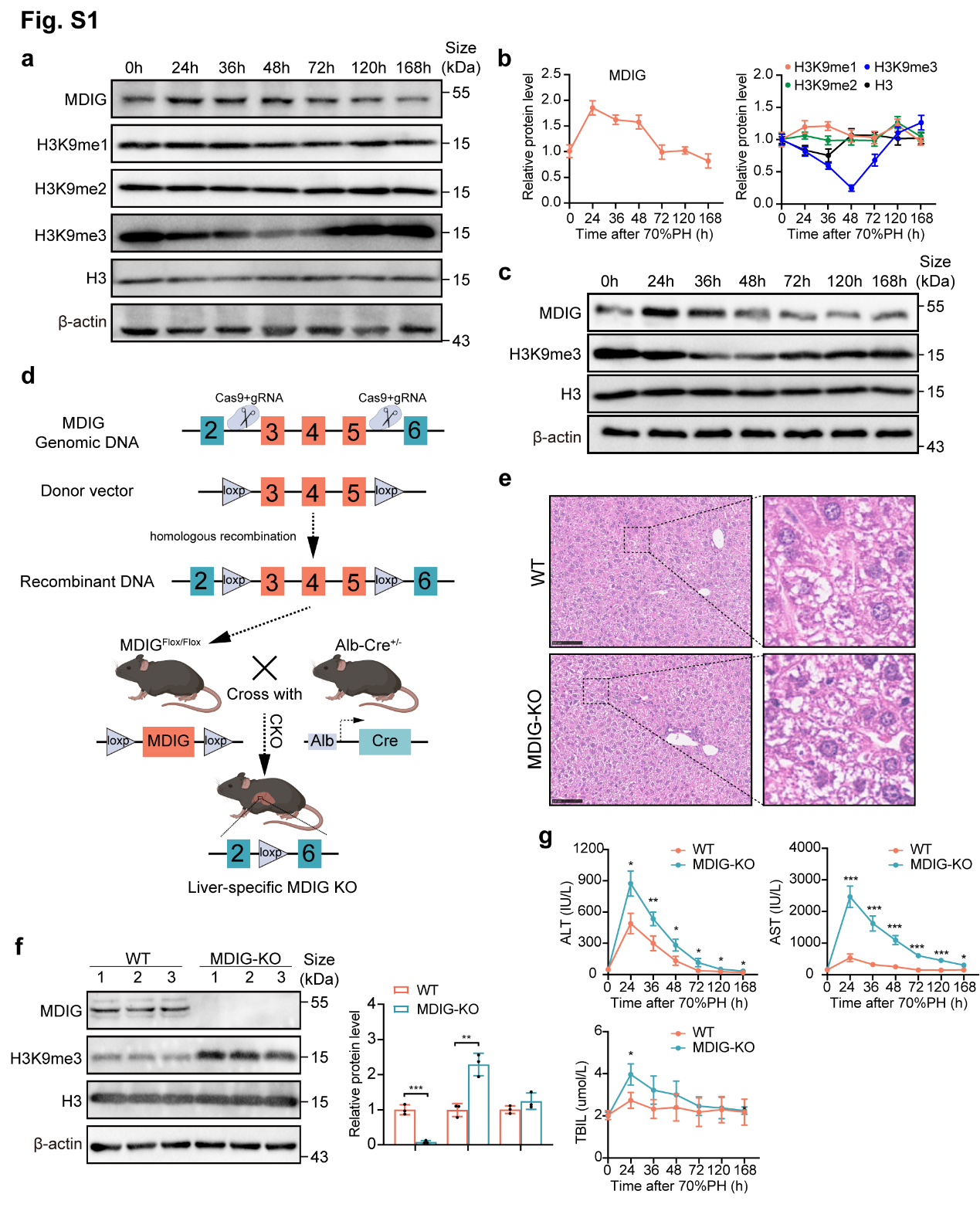
**

**Supplementary Fig. S1. Loss of MDIG impairs liver regeneration through modulating H3K9me3. (a, b)** Western blot analysis showing the expression of MDIG, H3K9me1, H3K9me2 and H3K9me3 in liver tissue lysates prepared from WT and MDIG-KO mice after PH. The 0 h time point was set as a control group after β-actin normalization. **(c)** Western blot analysis showing the expression of MDIG and H3K9me3 in liver tissue lysates prepared from WT and MDIG-KO mice after CCl_4_ challenge. **(d)** Schematic diagram of the strategy used to create liver-specific MDIG-KO mice. **(e)** Representative images of H&E stainings of wildtype liver and MDIG-KO liver showing no significant morphologic difference between the two mouse strains. **(f)** Western blot analysis showing the expression of MDIG, H3K9me1, H3K9me2 and H3K9me3 in liver tissue lysates prepared from WT and MDIG-KO mice after PH. The WT group was set as a control group after β-actin normalization. **(g)** Serum ALT, AST and TBIL levels at the indicated time points after PH. Data were shown as mean ± SD, n=3-5, unpaired Student’s t test, **P* < 0.05; ***P* < 0.01; ****P* < 0.001. Scale bars, 100μm.


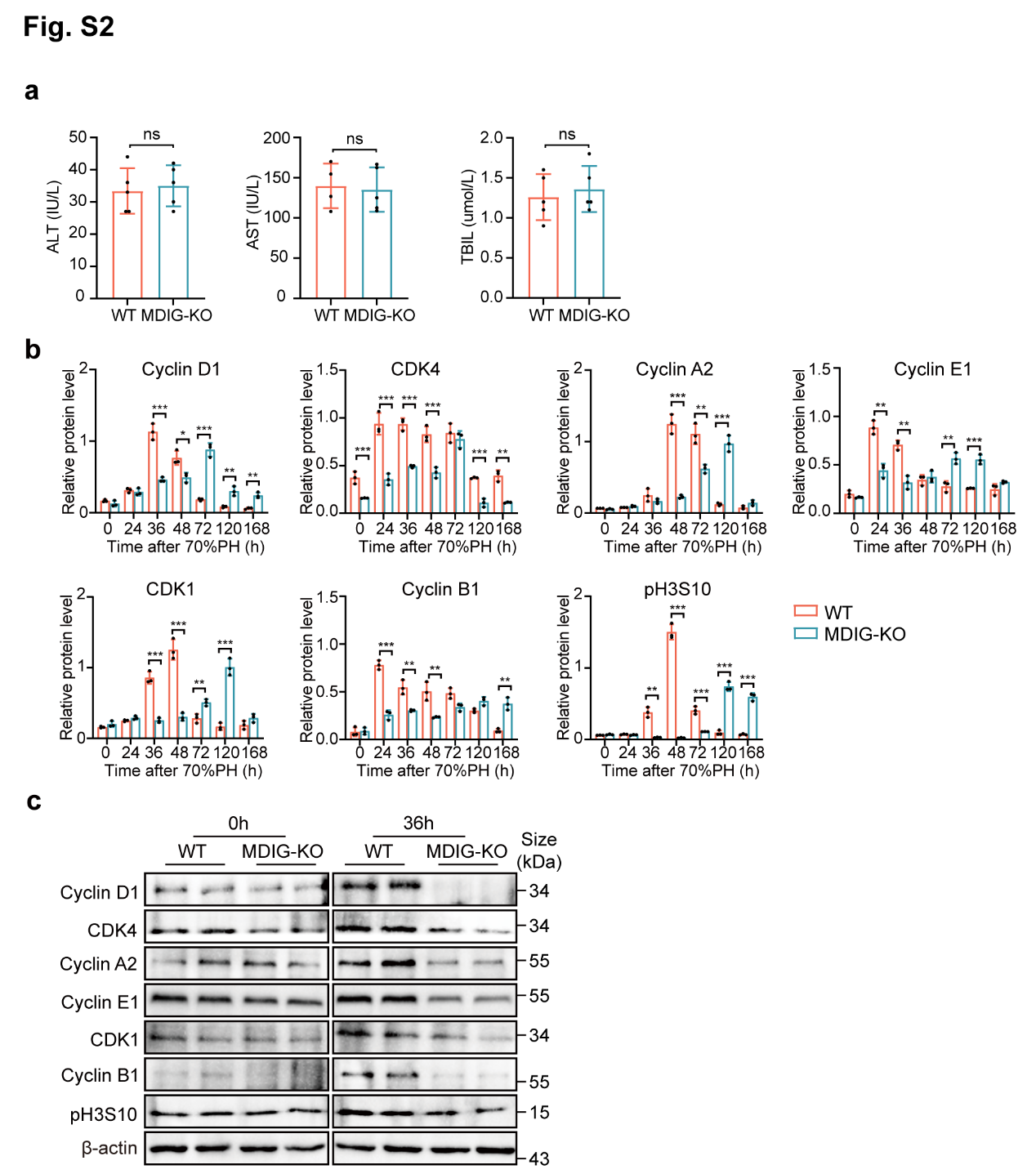


**Supplementary Fig. S2. MDIG knockout results in delayed hepatic recovery from PH by regulating cell cycle progression. (a)** Serum ALT, AST and TBIL levels at 14 days after PH. **(b)** Quantification data of the western blot results as related to Fig. 1a. The proteins levels are expressed as ratios to β-actin. **(c)** Western blot analysis on the cell cycle markers between WT and MDIG-KO livers at 0h and 36h after PH. Data were shown as mean ± SD, n=3-5, unpaired Student’s t test, **P* < 0.05; ***P* < 0.01; ****P* < 0.001.


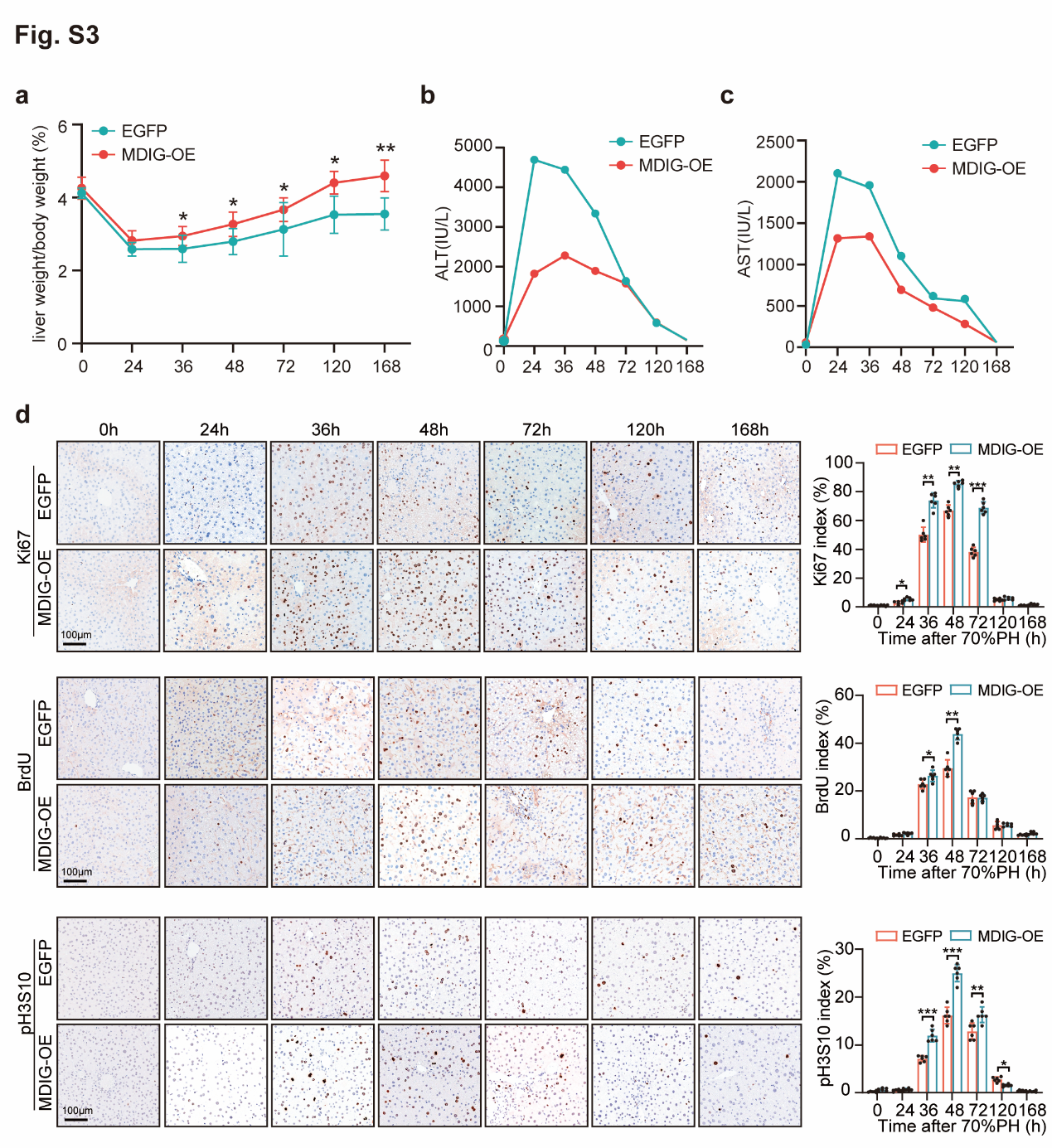


**Supplementary Fig. S3. MDIG overexpression facilitates liver regenerating following 70% PH. (a)** The ratio of liver weight/body weight at different time points after PH in MDIG overexpressed (MDIG-OE) mice and the control (EGFP) mice. **(b, c)** Serum ALT **(b)** and AST **(c)** levels at the indicated time points after PH. **(d)** Immunohistochemistry results for Ki67, BrdU and pH3S10 at different time points following PH. Data were shown as mean ± SD, n= 4-6, unpaired Student’s t test, **P* < 0.05; ***P* < 0.01; ****P* < 0.001. Scale bars, 100μm.

**
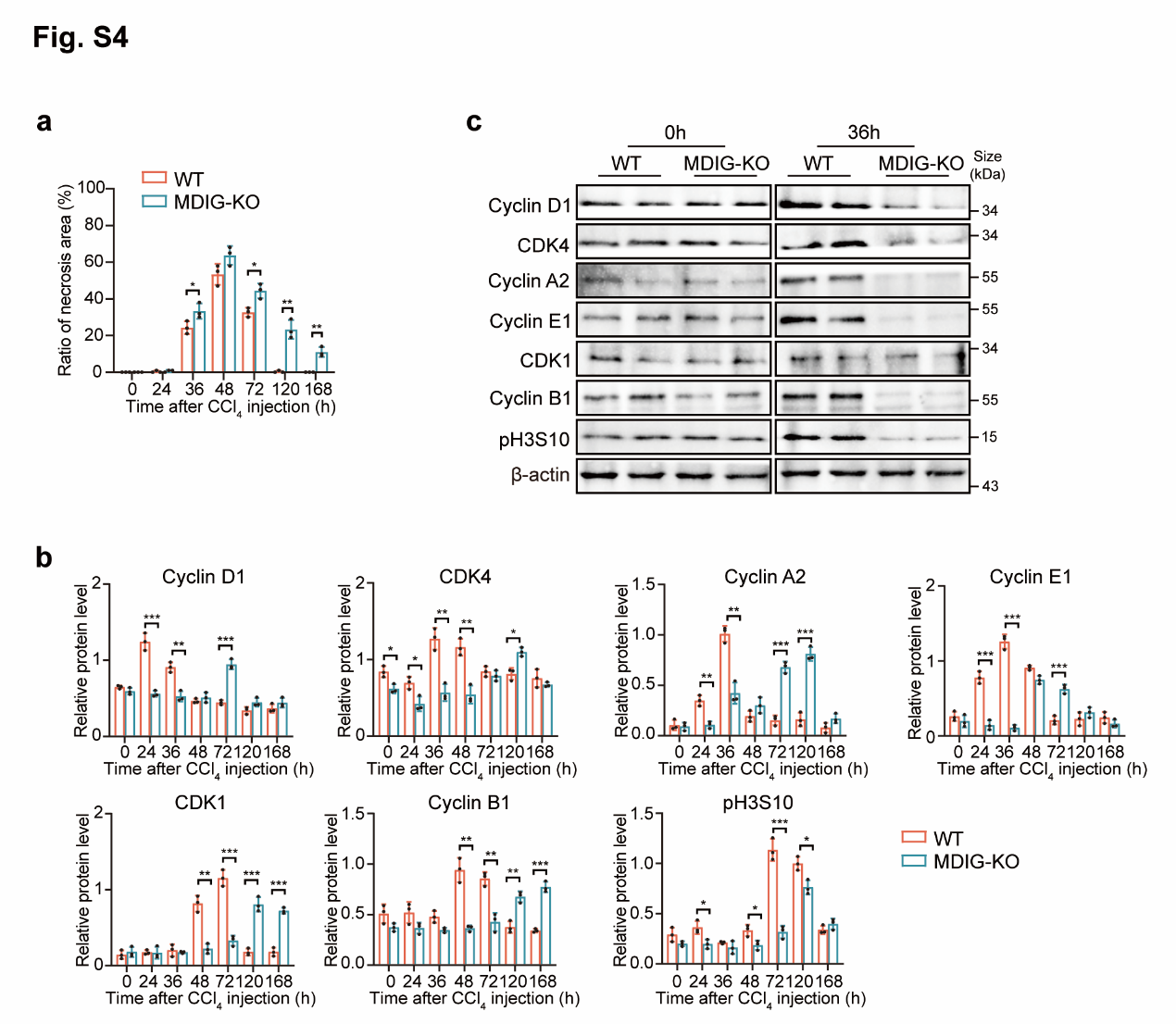
**

**Supplementary Fig. S4. MDIG knockout results in delayed hepatic recovery from CCl_4_ damage by regulating cell cycle progression. (a)** The percentages of hepatic necrotic area as related to Fig. 2a. **(b)** Quantification data of the western blot results as related to Fig. 2c. The proteins levels are expressed as ratios to β-actin. **(c)** Western blot analysis on the cell cycle markers between 0h and 36h after CCl_4_ challenge. Data were shown as mean ± SD, n=3-5, unpaired Student’s t test, **P* < 0.05; ***P* < 0.01; ****P* < 0.001.


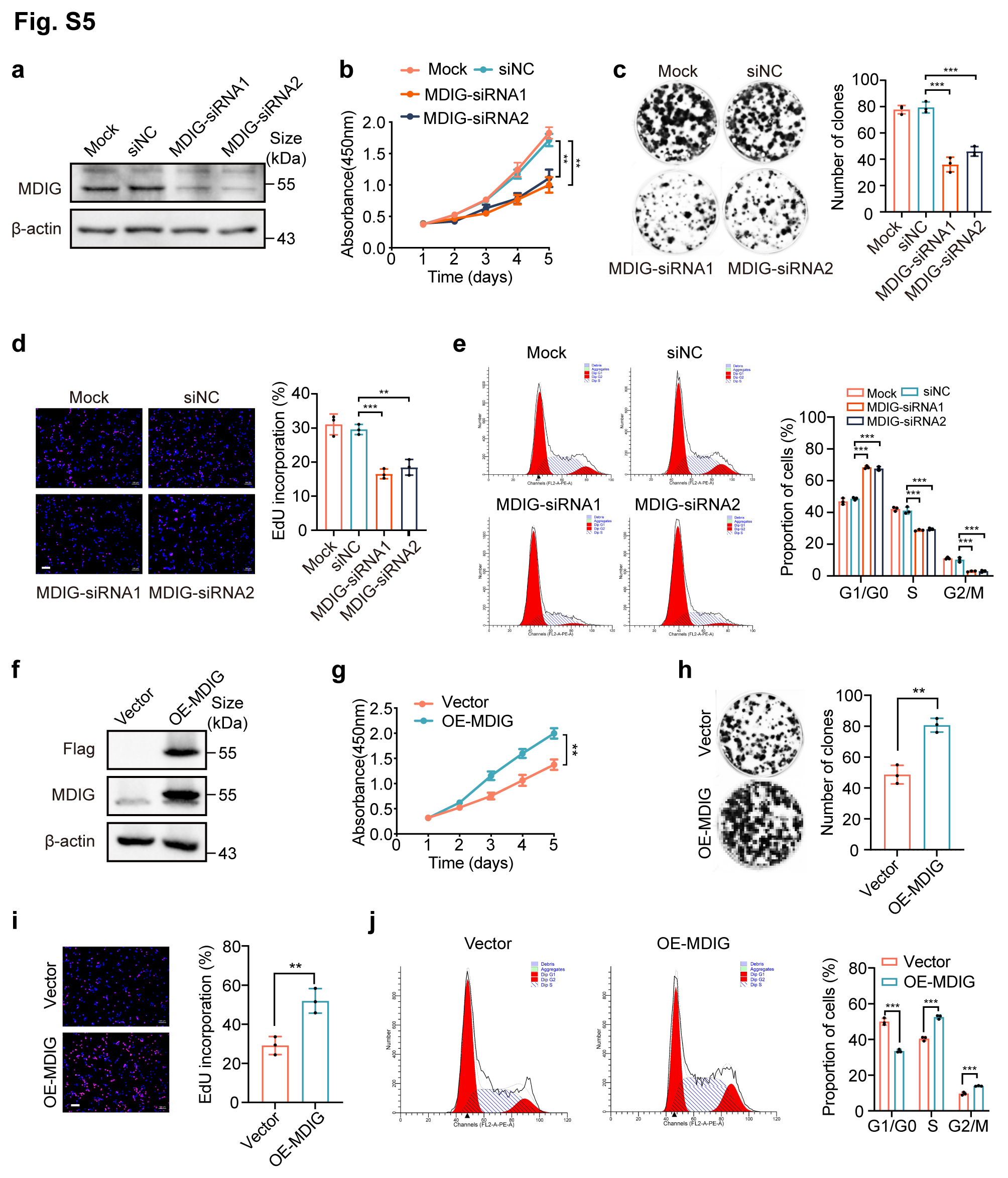


**Supplementary Fig. S5. MDIG promotes proliferation of Hepa1-6 cells *in vitro*.** **(a)** Western blot for the protein expression of MDIG after MDIG knockdown in Hepa1-6 cells. **(b)** CCK8 assays revealed cell proliferation capacity after silencing MDIG. **(c)** Colony formation assays revealed colony formation activity after silencing MDIG. **(d)** EdU immunofluorescence assays revealed cell proliferation capacity after silencing MDIG. **(e)** Cell cycle assays showing effects of MDIG knockdown on the proliferation of Hepa1-6 cells. **(f)** Western blot for the protein expression of MDIG after MDIG overexpression in Hepa1-6 cells. **(g)** CCK8 assays revealed cell proliferation capacity after overexpressing MDIG. **(h)** Colony formation assays revealed colony formation activity after overexpressing MDIG. **(i)** EdU immunofluorescence assays revealed cell proliferation capacity after overexpressing MDIG. **(j)** Cell cycle assays showing effects of MDIG overexpression on the proliferation of Hepa1-6 cells. Data were shown as mean ± SD, unpaired Student’s t test, **P* < 0.05; ***P* < 0.01; ****P* < 0.001. Scale bars, 100μm.


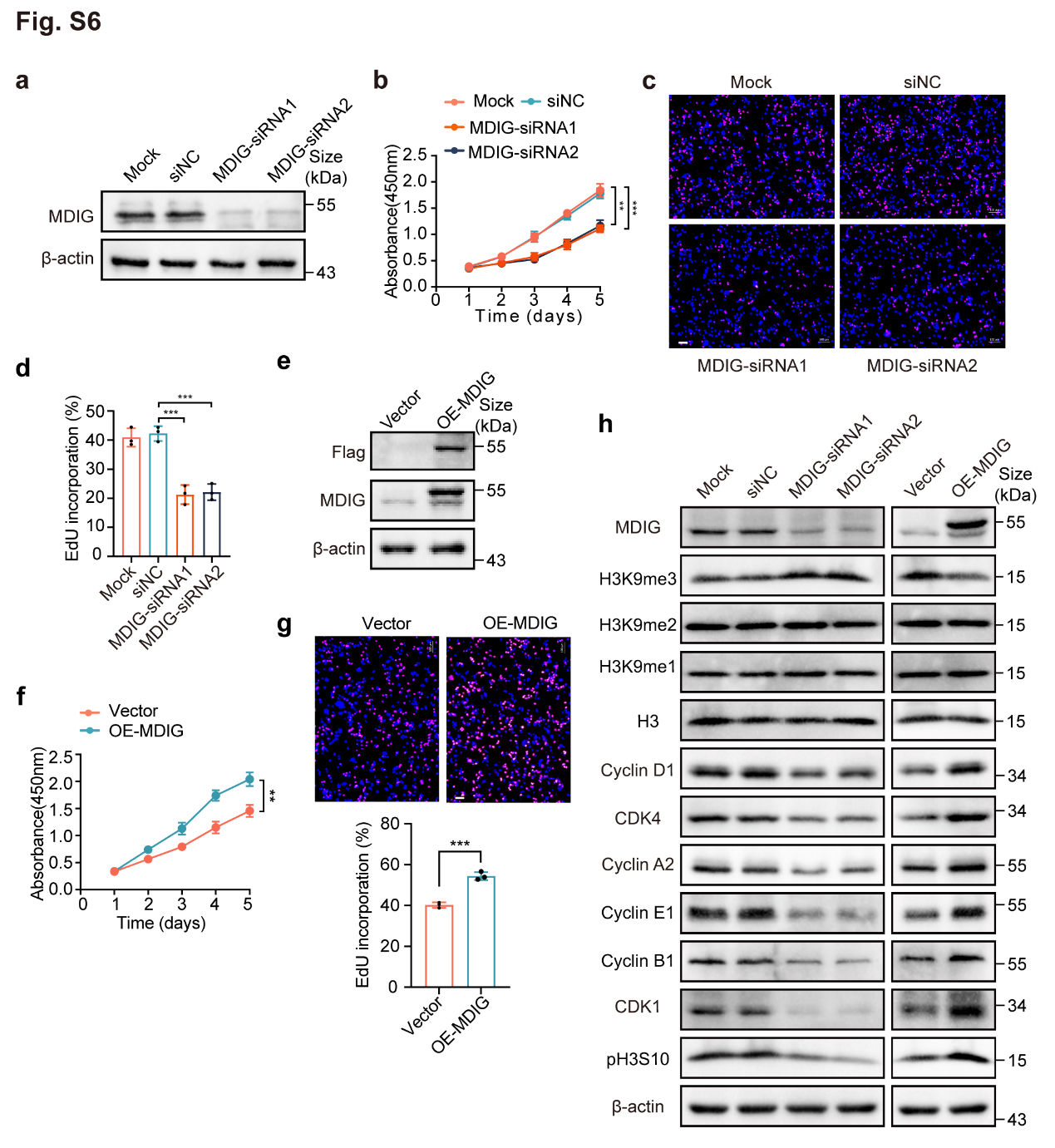


**Supplementary Fig. S6. MDIG promotes proliferation of AML12 cells *in vitro*. (a)** Western blot for the protein expression of MDIG after MDIG knockdown in AML12 cells. **(b)** CCK8 assays revealed cell proliferation capacity after silencing MDIG. **(c)** EdU immunofluorescence assays revealed cell proliferation capacity after silencing MDIG. **(d)** Cell cycle assays showing effects of MDIG knockdown on the proliferation of AML12 cells. **(e)** Western blot for the protein expression of MDIG after MDIG overexpression in AML12 cells. **(f)** CCK8 assays revealed cell proliferation capacity after overexpressing MDIG. **(g)** EdU immunofluorescence assays revealed cell proliferation capacity after overexpressing MDIG. **(h)** Western blot analysis of cell cycle markers after MDIG knockdown or overexpression *in vitro*. Data were shown as mean ± SD, unpaired Student’s t test, **P* < 0.05; ***P* < 0.01; ****P* < 0.001. Scale bars, 100μm.


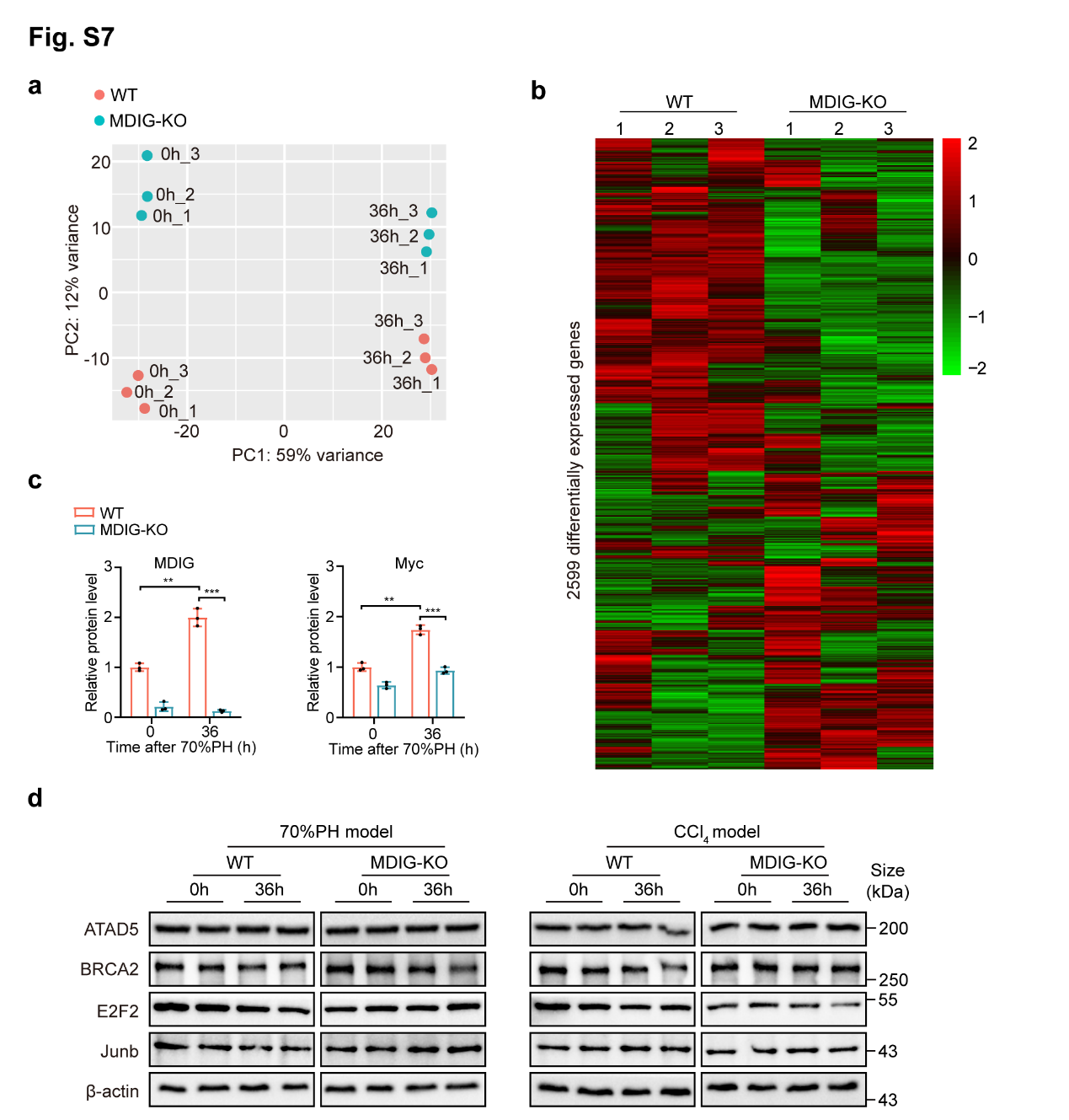


**Supplementary Fig. S7. Analysis of the WT and MDIG-KO livers during liver regeneration. (a)** The principal component analysis of the RNA-seq results. **(b)** The heatmap of 2599 differentially expressed genes in WT and MDIG-KO livers at 0 h after PH. **(c)** Quantification data of the western blot results as related to Fig. 3d. The WT group at 0 h time point was set as a control group after β-actin normalization. **(d)** Western blot analysis of the cell cycle regulators at 0h and 36h after PH or CCl_4_ treatment.

**
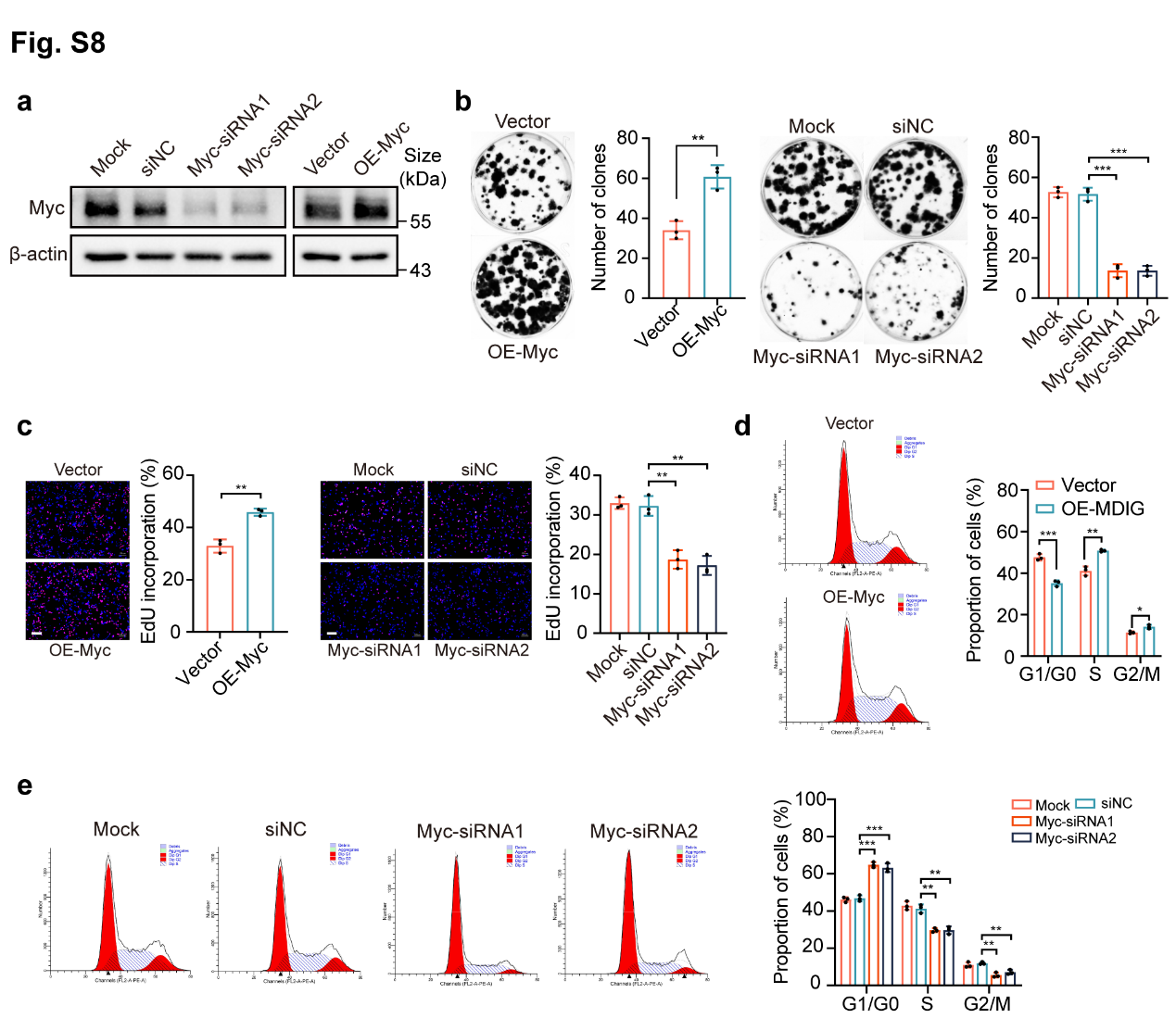
**

**Supplementary Fig. S8. Myc regulates cell growth *in vitro*.** **(a)** Western blot analysis showing the expression of Myc after silencing or overexpressing Myc in Hepa1-6 cells. **(b)** Colony formation assays revealed cell proliferation capacity after overexpressing Myc (left) or silencing Myc (right) in Hepa1-6 cells. **(c)** EdU immunofluorescence assays revealed cell proliferation capacity after overexpressing Myc (left) or silencing Myc (right) in Hepa1-6 cells. **(d, e)** Cell cycle assays showing effects of MDIG overexpression **(d)** or knockdown **(e)** on the proliferation of Hepa1-6 cells. Data were shown as mean ± SD, unpaired Student’s t test, **P* < 0.05; ***P* < 0.01; ****P* < 0.001. Scale bars, 100μm.


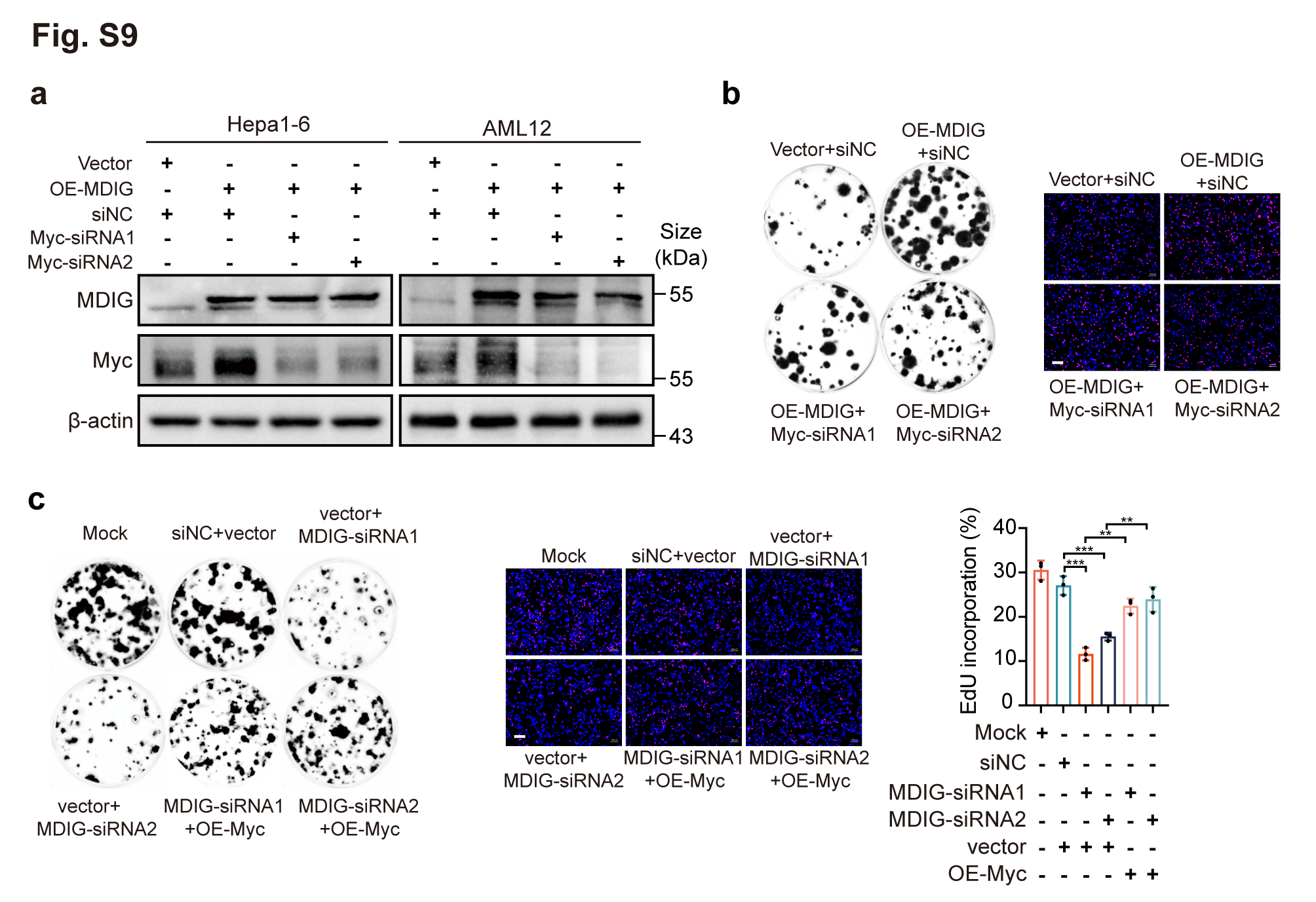


**Supplementary Fig. S9. MDIG promotes cell proliferation by enhancing Myc expression.** **(a)** Western blot analysis showing the expression of MDIG and Myc after silencing Myc in MDIG-overexpressing Hepa1-6 and AML12 cells. **(b)** Representative images of colony formation assays (left) and EdU immunofluorescence assays (right) after silencing Myc in MDIG-overexpressing Hepa1-6 cells. **(c)** Colony formation assays (left) and EdU immunofluorescence assays (right) revealed cell proliferation capacity after overexpressing Myc in MDIG-silencing Hepa1-6 cells.

**
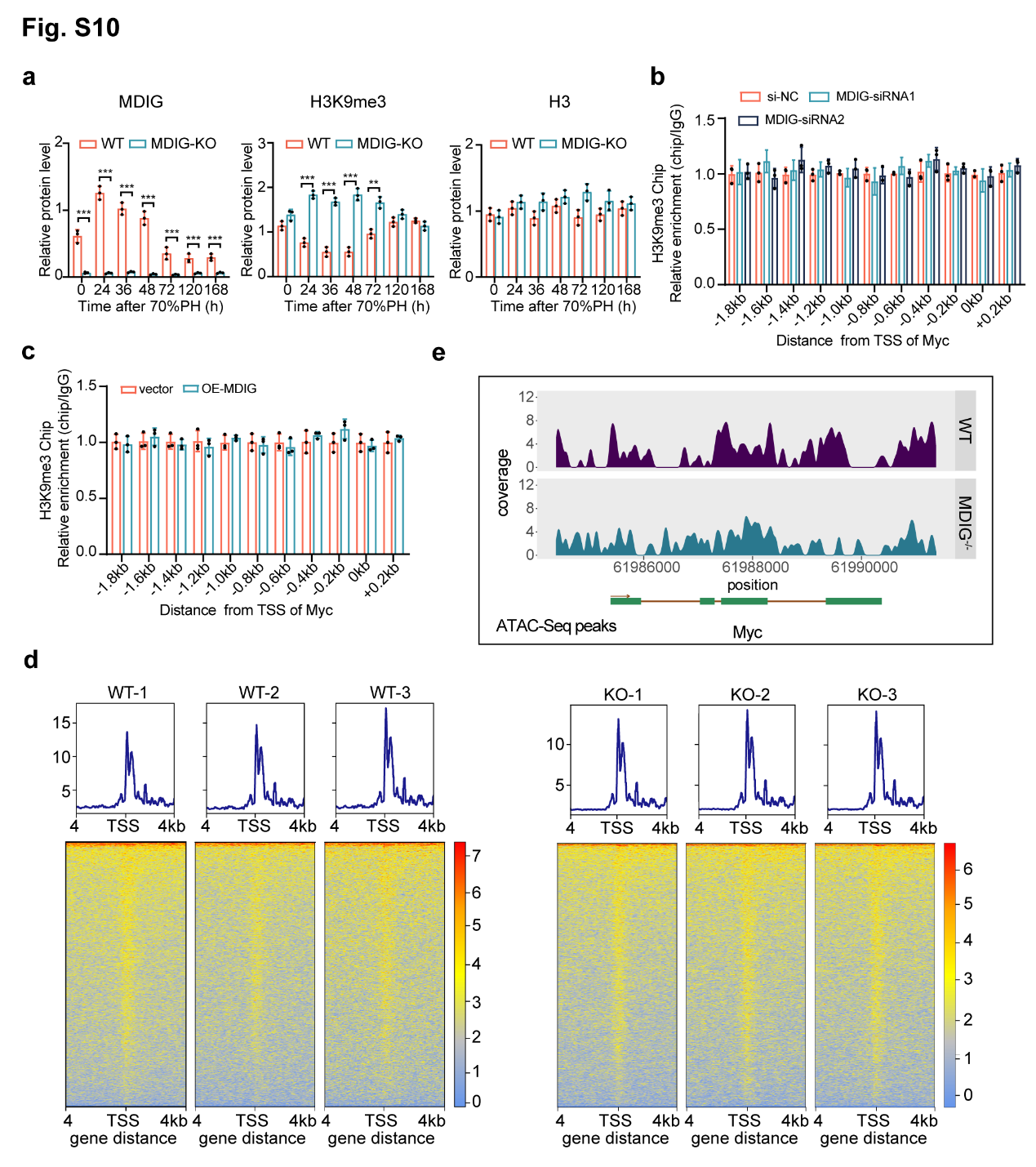
**

**Supplementary Fig. S10. MDIG ablation reduces chromatin accessibility for OTX2 by increasing the H3K9 methylation of its promoter. (a)** Quantification data of the western blot results as related to Fig. 4a. The proteins levels are expressed as ratios to β-actin. **(b)** ChIP-qPCR was conducted to evaluate the enrichment of H3K9me3 in different promoter regions of Myc in Hepa1-6 cells after silencing MDIG. **(c)** ChIP-qPCR was conducted to evaluate the enrichment of H3K9me3 in different promoter regions of Myc in Hepa1-6 cells after overexpressing MDIG (OE-MDIG). **(d)** Heatmaps of ATAC-seq using livers from WT and MDIG-KO mice at 36 h after PH showing open chromatin regions focused on the transcription start site (TSS). **(e)** Genome browser view showing ATAC-seq signals around the Myc loci.


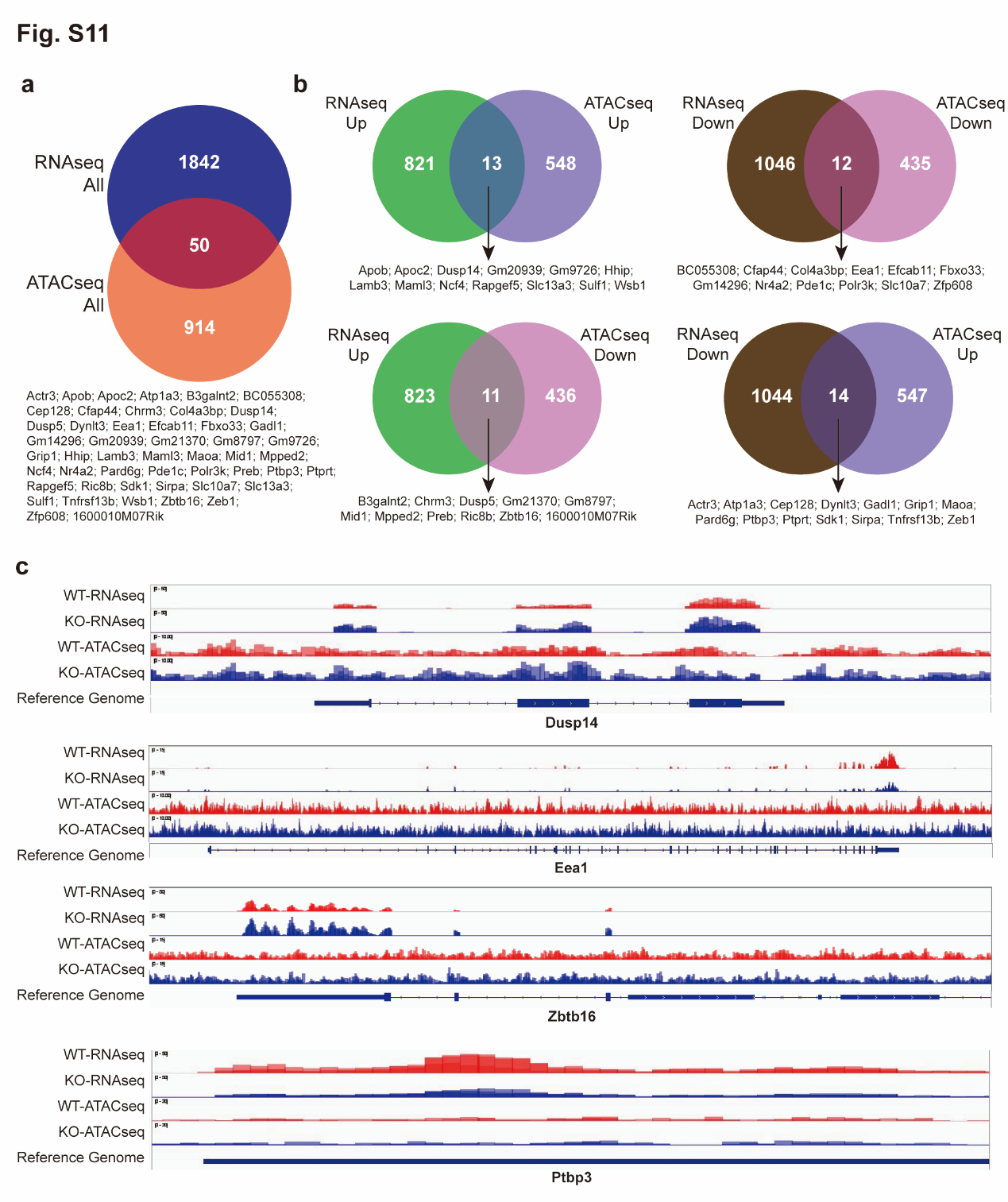


**Supplementary Fig. S11. Integration analysis on the RNA-seq and ATAC-seq results. (a)** Venn diagram showing overlapping of the differentially expressed genes as identified from RNA-seq and the genes with altered chromatin accessibility as identified from ATAC-seq at 36h after PH. The official names of the 50 overlapped genes are provided. **(b)** Subgroup analysis identified four groups of the genes based on their transcriptional expression and chromatin accessibility. Group 1: Genes with increased gene expression (RNA-seq Up) and increased chromatin accessibility (ATAC-seq Up); Group 2: Genes with decreased gene expression (RNA-seq down) and decreased chromatin accessibility (ATAC-seq down); Group3: Genes with increased gene expression (RNA-seq up) but decreased chromatin accessibility (ATAC-seq down); Group 4: Genes with decreased gene expression (RNA-seq down) but increased chromatin accessibility (ATAC-seq Up). The official names of the overlapped genes in each group are provided below the Venn graphs. **(c)** Representative images of genome browser view showing RNA expression information and ATAC-seq signals around the genes of interest loci (Dusp14 in group 1; Eea1 in group 2; Zbtp16 in group 3 and Ptbp3 in group4).


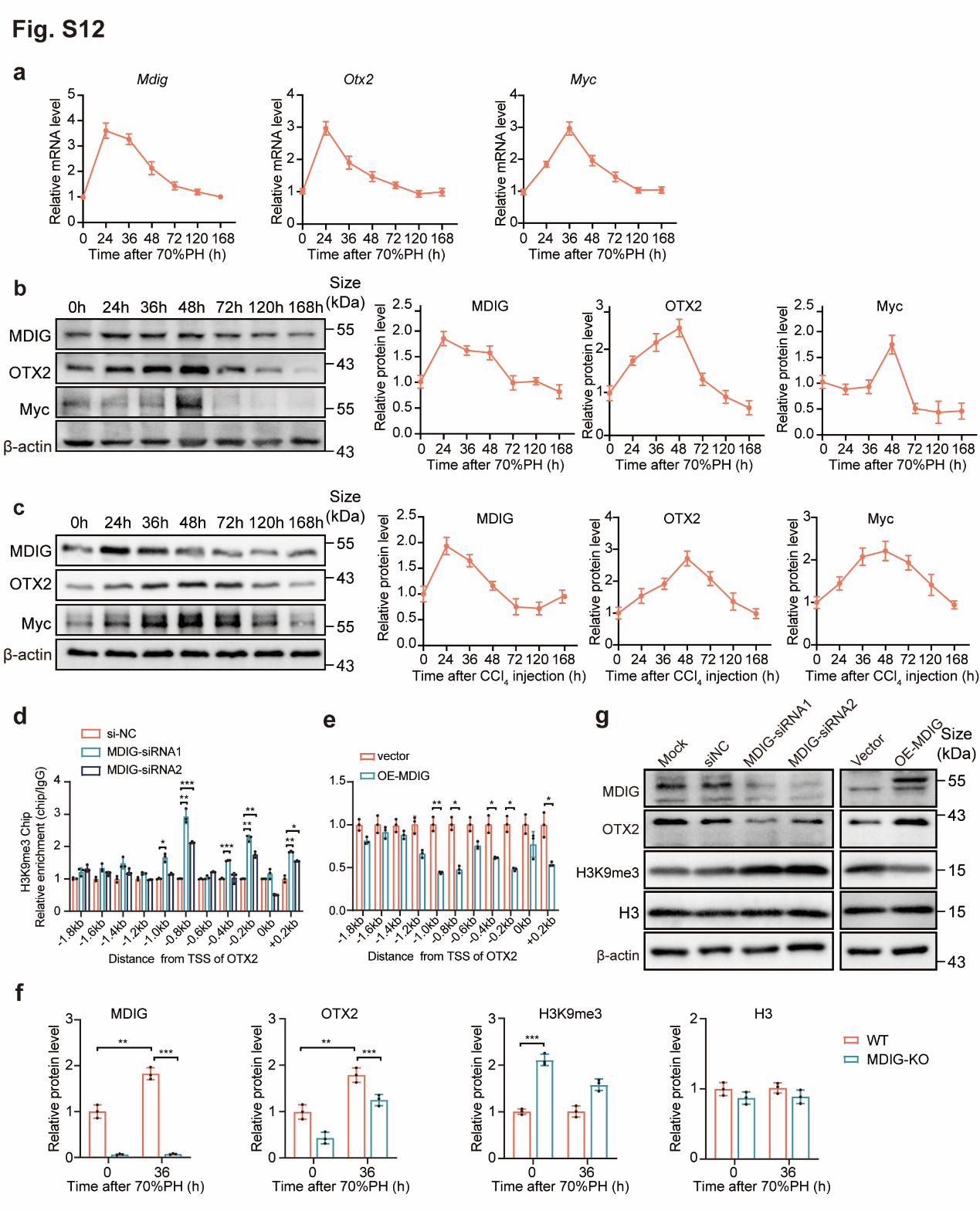


**Supplementary Fig. S12. Dynamic changes of MDIG, OTX2 and MYC during liver regeneration. (a)** Expression levels of *Mdig, Otx2* and *Myc* mRNA at different time points after PH. **(b)** Expression levels of MDIG, OTX2 and Myc proteins at different time points after PH. The 0 h time point was set as a control group after β-actin normalization. **(c)** Expression levels of MDIG, OTX2 and Myc proteins at different time points after CCl_4_ treatment. The 0 h time point was set as a control group after β-actin normalization. **(d, e)** ChIP-qPCR was conducted to evaluate the enrichment of H3K9me3 at different promoter regions of OTX2 in Hepa1-6 cells after silencing **(d)** or overexpressing **(e)** MDIG. **(f)** Quantification data of the western blot results as related to Fig. 4i. The WT group at 0 h time point was set as a control group after β-actin normalization. **(g)** Western blot for indicated protein expression after MDIG knockdown or overexpression in Hepa1-6 cells.

**
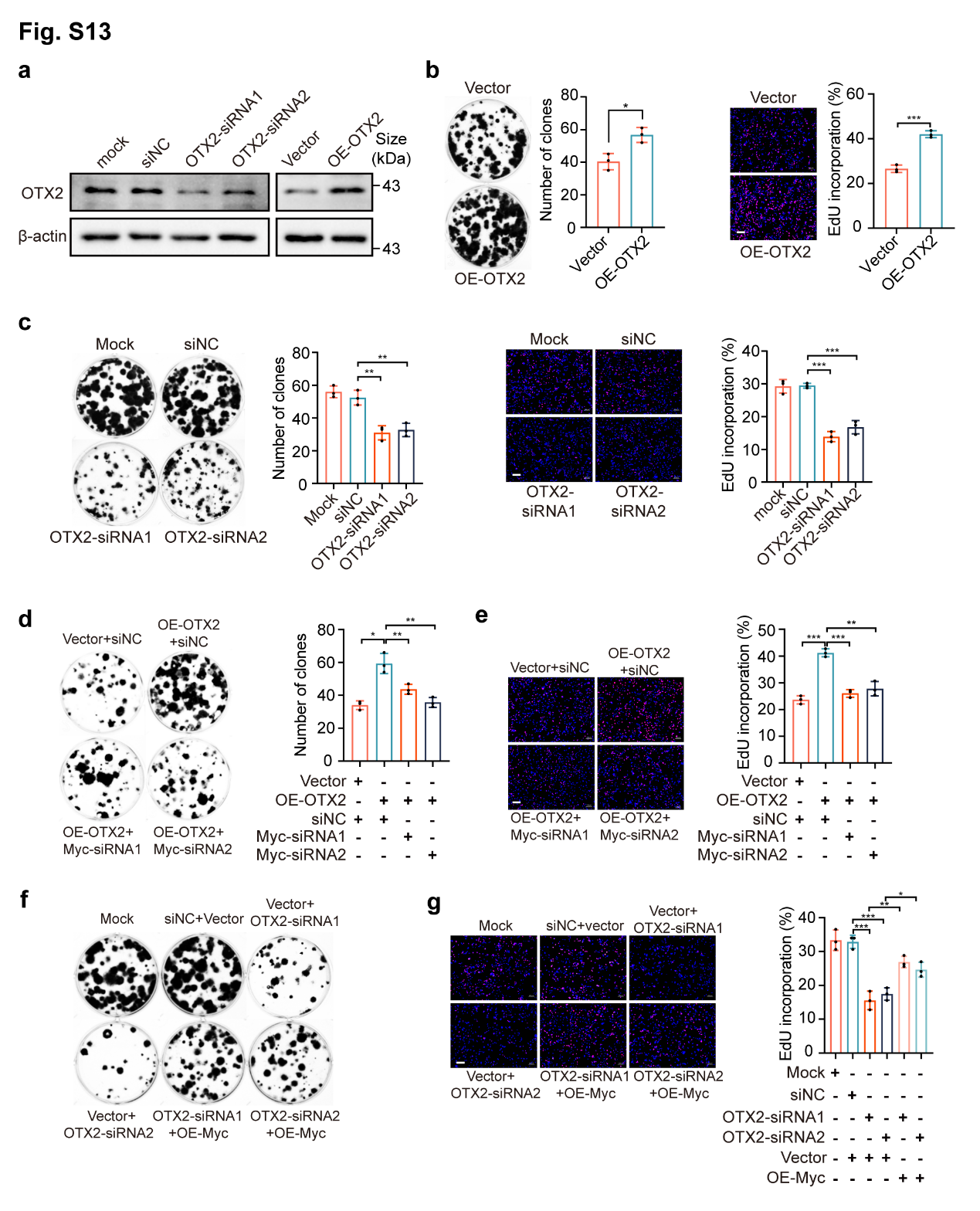
**

**Supplementary Fig. S13. OTX2 promotes cell proliferation by enhancing Myc expression. (a)** Western blot analysis showing the expression of OTX2 after silencing or overexpressing OTX2 in Hepa1-6 cells. **(b)** Colony formation assays (left) and EdU immunofluorescence assays (right) revealed cell proliferation capacity after overexpressing OTX2 in Hepa1-6 cells. **(c)** Colony formation assays (left) and EdU immunofluorescence assays (right) revealed cell proliferation capacity after silencing OTX2 in Hepa1-6 cells. **(d, e)** Representative images of colony formation assays **(d)** and EdU immunofluorescence assays **(e)** after silencing Myc in OTX2-overexpressing Hepa1-6 cells. **(f)** Colony formation assays revealed cell proliferation capacity after overexpressing Myc in OTX2-silencing Hepa1-6 cells. **(g)** EdU immunofluorescence assays revealed cell proliferation capacity after overexpressing Myc in OTX2-silencing Hepa1-6 cells. Data were shown as mean ± SD, unpaired Student’s t test, **P* < 0.05; ***P* < 0.01; ****P* < 0.001. Scale bars, 100μm.


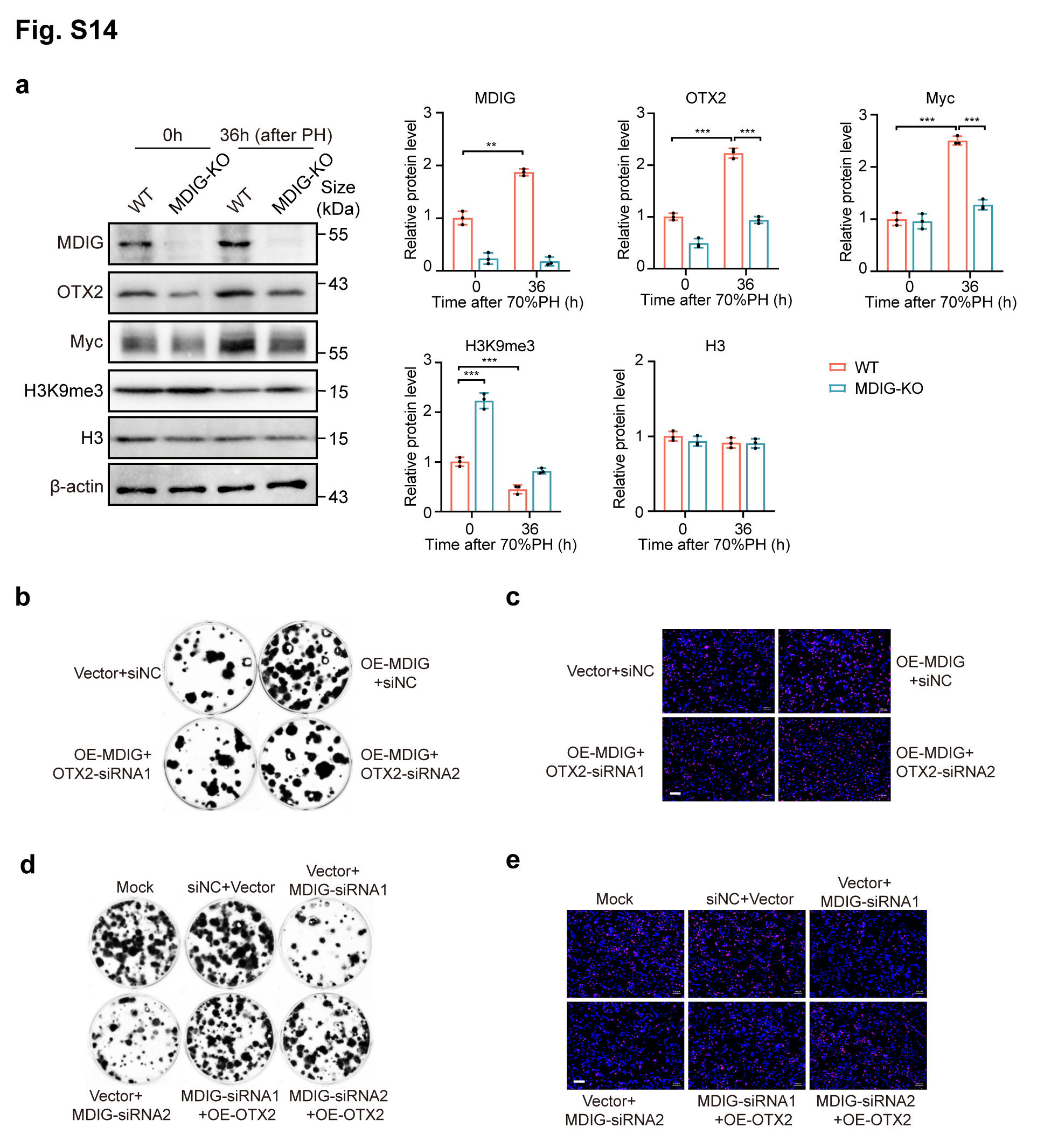


**Supplementary Fig. S14. MDIG promotes Myc expression by upregulation of OTX2. (a)** Western blot for the indicated protein expression in liver tissue lysates prepared from WT and MDIG-KO mice at different times after PH. The WT group at 0 h time point was set as a control group after β-actin normalization. **(b, c)** Colony formation assays **(b)** and EdU immunofluorescence assays **(c)** revealed cell proliferation capacity after silencing OTX2 in MDIG-overexpressing Hepa1-6 cells. **(d, e)** Representative images of colony formation assays **(d)** and EdU immunofluorescence assays **(e)** revealed cell proliferation capacity after overexpressing OTX2 in MDIG-silencing Hepa1-6 cells. Scale bars, 100μm.


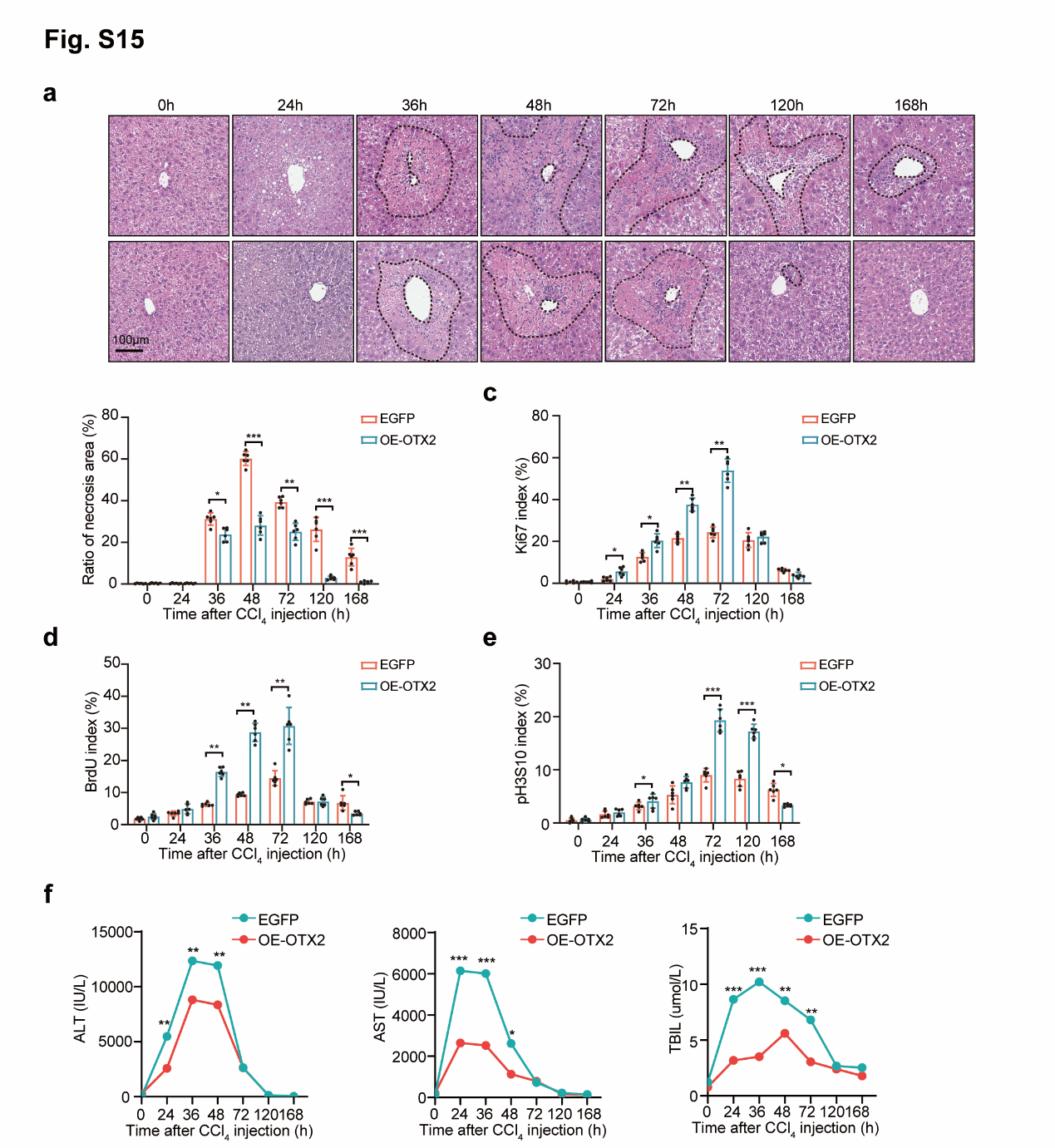


**Supplementary Fig. S15. OTX2 overexpression facilitates rapid hepatic recovery in CCl_4_ treated MDIG knockout mice. (a)** H&E staining shows liver repair was dramatically impaired in EGFP transfected MDIG-KO liver. In contrast, OTX2 overexpression (OE-OTX2) promotes liver recovery in the MDIG-KO livers. The necrotic areas were circled with dotted lines. **(b)** The percentages of hepatic necrotic area at the indicated time points in both groups. **(c-e)** Percentages of Ki67 **(c)**, BrdU **(d)** and pH3S10 (**e)** immunoreactive positive cells at different time points following CCl_4_ challenge. **(f)** Serum ALT, AST and TBIL levels at the indicated time points after CCl_4_ challenge. Data were shown as mean ± SD, n=3-5, unpaired Student’s t test, **P* < 0.05; ***P* < 0.01; ****P* < 0.001. Scale bars, 100μm.


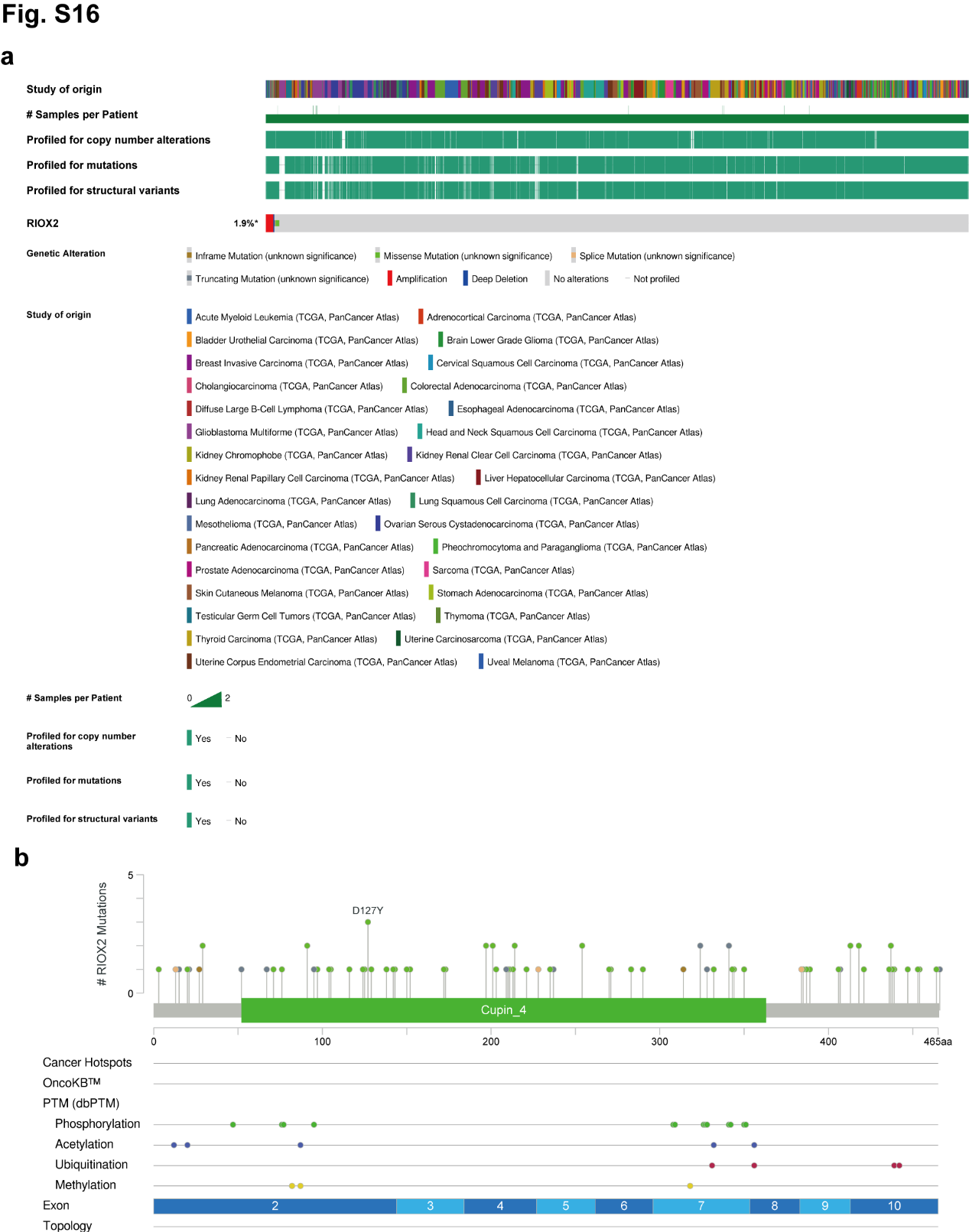


**Supplementary Fig. S16. Mutation signature of MDIG in TCGA PanCancer Atlas. (a)** Heatmap showing the percentages of MDIG (also named as RIOX2) mutated samples across the human cancer samples. A total of 10950 samples from 32 types of cancers were profiled and 209 samples (~1.9%) samples harbored MDIG genomic alterations. The types of genomic alterations included amplification, missense mutation, deep deletion, truncating mutation, splice mutation and inframe mutation. **(b)** Lollipop plot showing the mutation signature in MDIG (RIOX2) genome. A total of 84 (0.7%) somatic mutations were found. The results were analyzed using the online cBioPortal dataset (https://www.cbioportal.org).

_
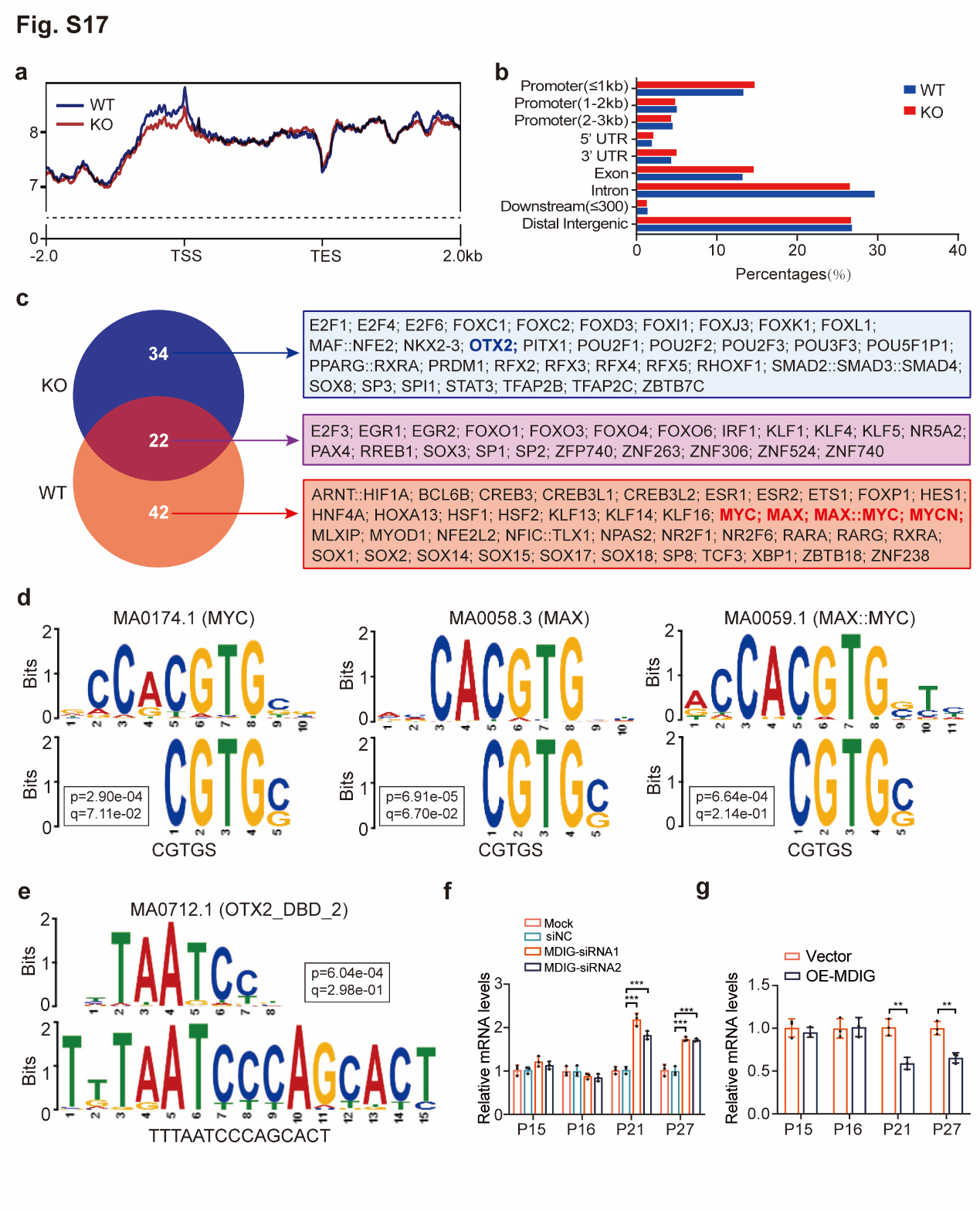
_

**Supplementary Fig. S17. Whole genome analysis of H3K9me3 before and after MDIG deletion by using Cleavage Under Targets and Tagmentation (CUT&Tag). (a)** Distribution of read counts upstream and downstream of the gene body as detected by CUT&Tag for the H3K9me3 histone modification. Scale regions were 2,000 bp upstream of the translation starting site (TSS)- 2,000 bp downstream of the translation end site (TES). **(b)** Genomic distribution of H3K9me3 signal intensities based on CUT&Tag profiling in the WT and MDIG-KO livers. The results indicated that H3K9me3 shifted to access the 0-1kb promoter region and Exon upon MDIG deletion. **(c)** Significant motif of peaks in the MDIG-KO and WT livers. Significantly different peaks were identified with M-A plot and then were analyzed by MEME and DREME software followed by alignment to the motif database. The results suggested that MDIG deletion significantly affected the H3K9me3 modification on transcriptional factors of OTX2 without affecting MYC (or MAX, MAX::MYC). **(d)** Sequence logo of the known motifs of MYC (or MAX, MAX::MYC) and the identified sequence of H3K9me3 binding motifs in the WT livers. The p values and q values were indicated in the images. **(e)** Sequence logo of the known motifs of OTX2 and the identified sequence of H3K9me3 binding motifs in the KO livers. The p value and q value were indicated in the image. **(f, g)** mRNA levels of cell cycle inhibitors in the MDIG silenced **(f)** and MDIG overexpressed **(g)** AML12 cells. Data were shown as mean ± SD, n=3-5, unpaired Student’s t test, **P* < 0.05; ***P* < 0.01; ****P* < 0.001. Scale bars, 100μm.


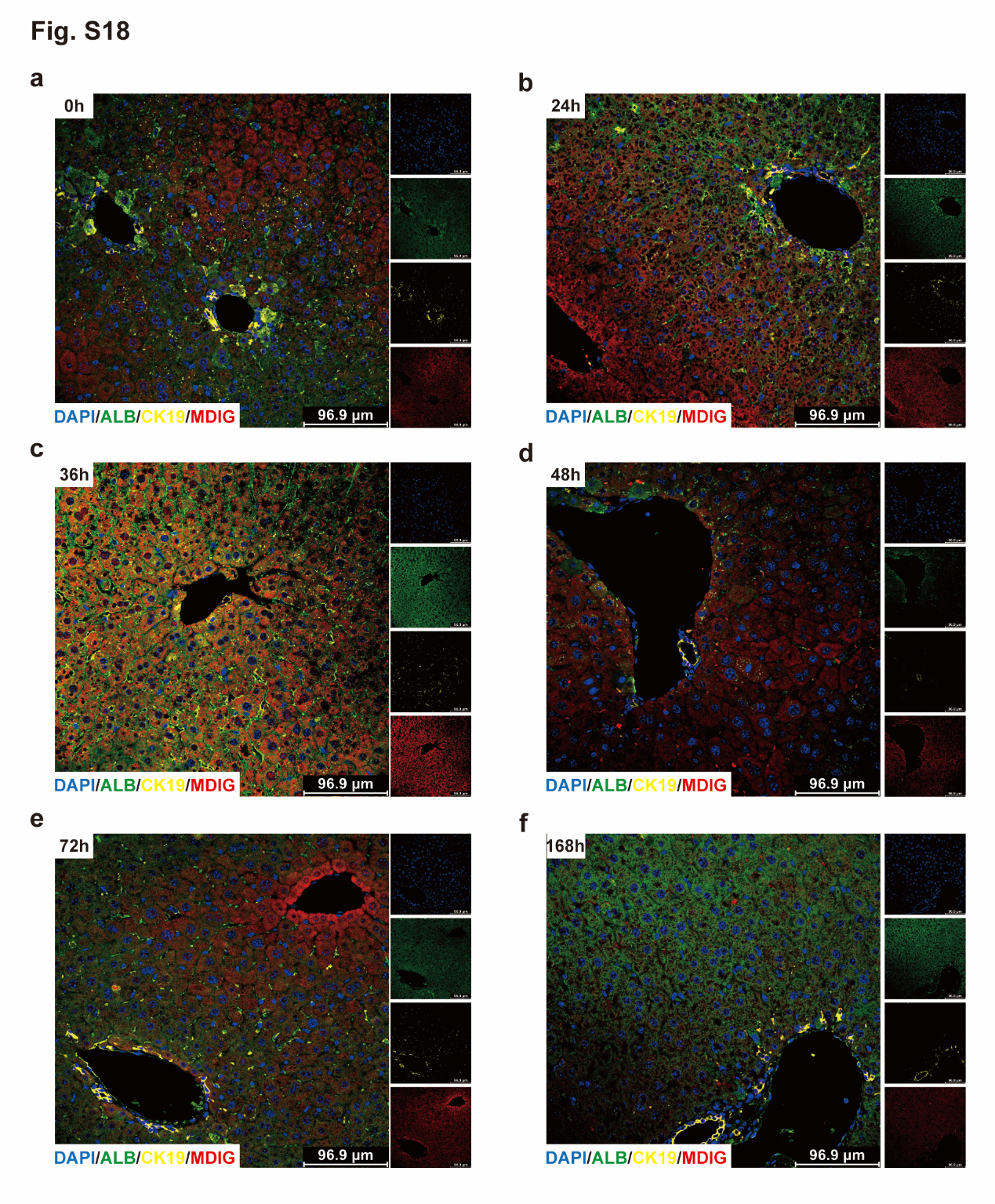


**Supplementary Fig. S18. Expression of MDIG in hepatocytes and cholangiocytes during liver regeneration. (a-f)** Co-immunofluorescence stainings of MDIG (red), ALB (biomarker for hepatocytes, green) and CK19 (biomarker for cholangiocytes, yellow) and nucleus marker DAPI (blue) on the liver sections from the indicated time points after PH. MDIG maintains a medium level in the quiescent liver cells **(a)** at 0h after PH, with the majority express in the hepatocytes around the peri-central regions. Levels of MDIG remains increasing along with the liver regeneration till 48h after PH. During this period, co-localization of MDIG and ALB or CK19 could both be observed. Expression of MDIG tends to expend from the peri-central regions to the peri-portal regions **(b-d)**. Then, levels of MDIG gradually decr­­­eases to the levels lower than 0h. During this period, co-localization of MDIG and ALB or CK19 could also be observed. However, the expression of levels of MDIG in the peri-central regions remains higher than the peri-portal regions **(e, f)**.


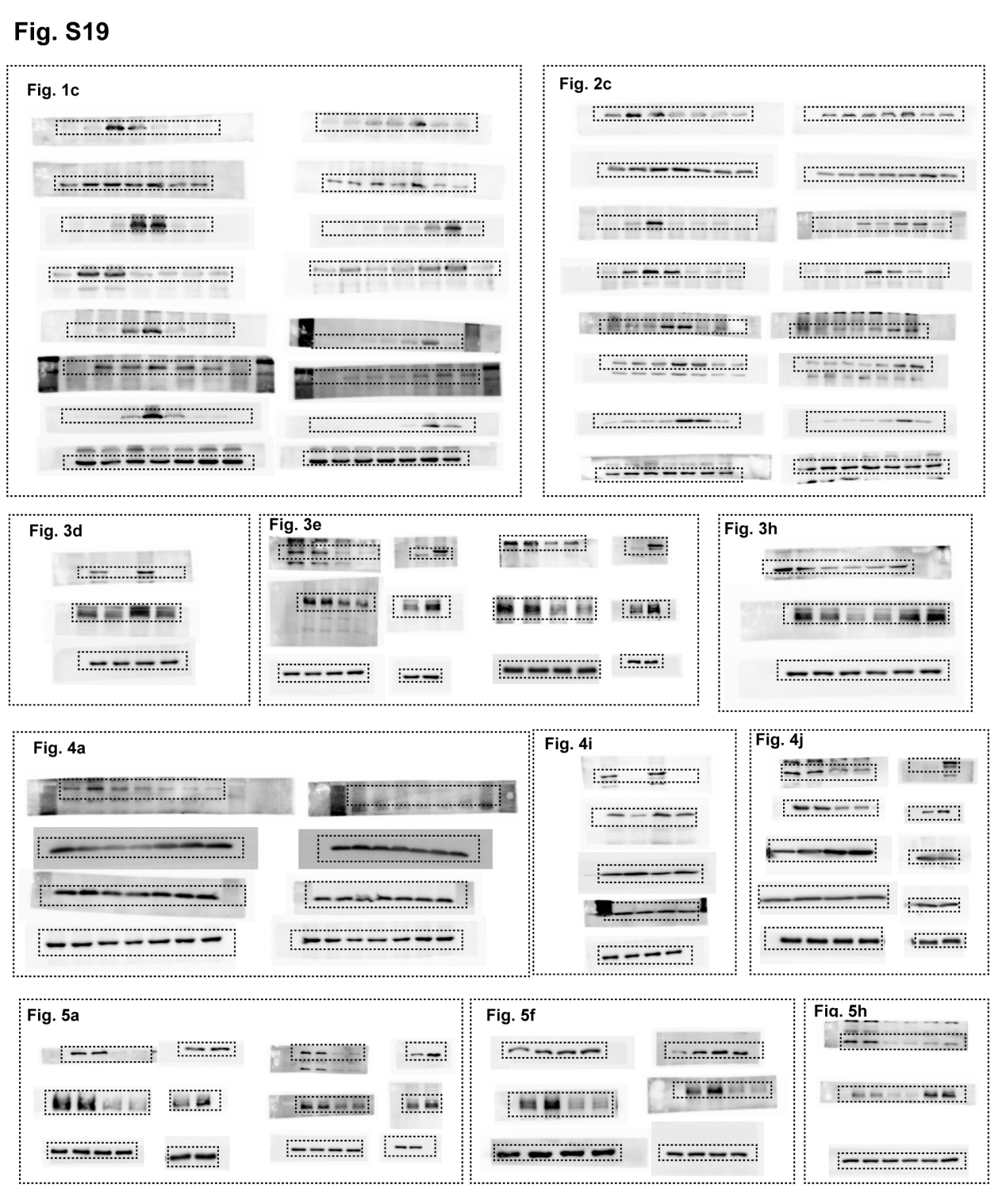


**Supplementary Fig. S19. The original and uncropped films of Western blots.**


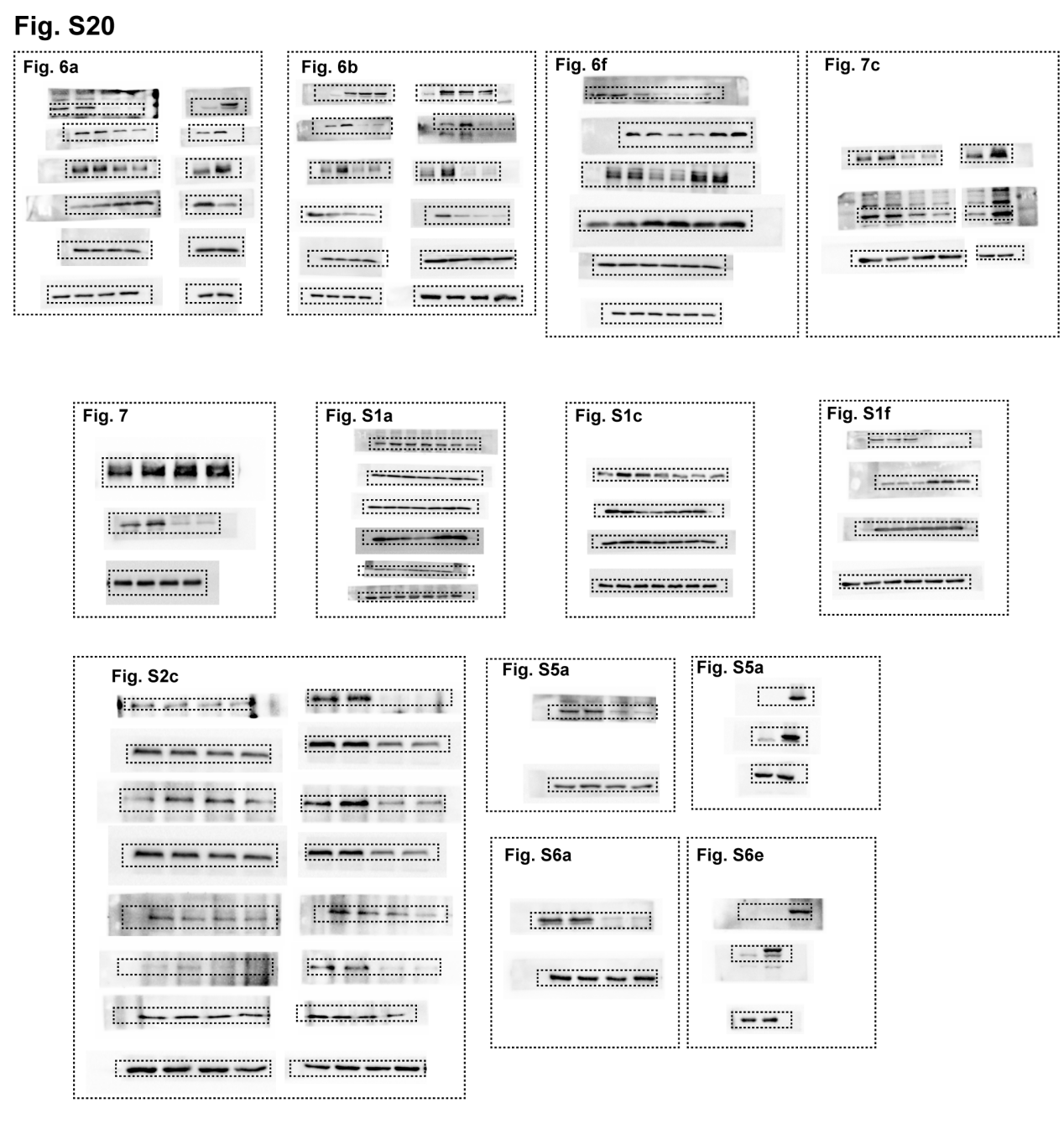


**Supplementary Fig. S20. The original and uncropped films of Western blots.**


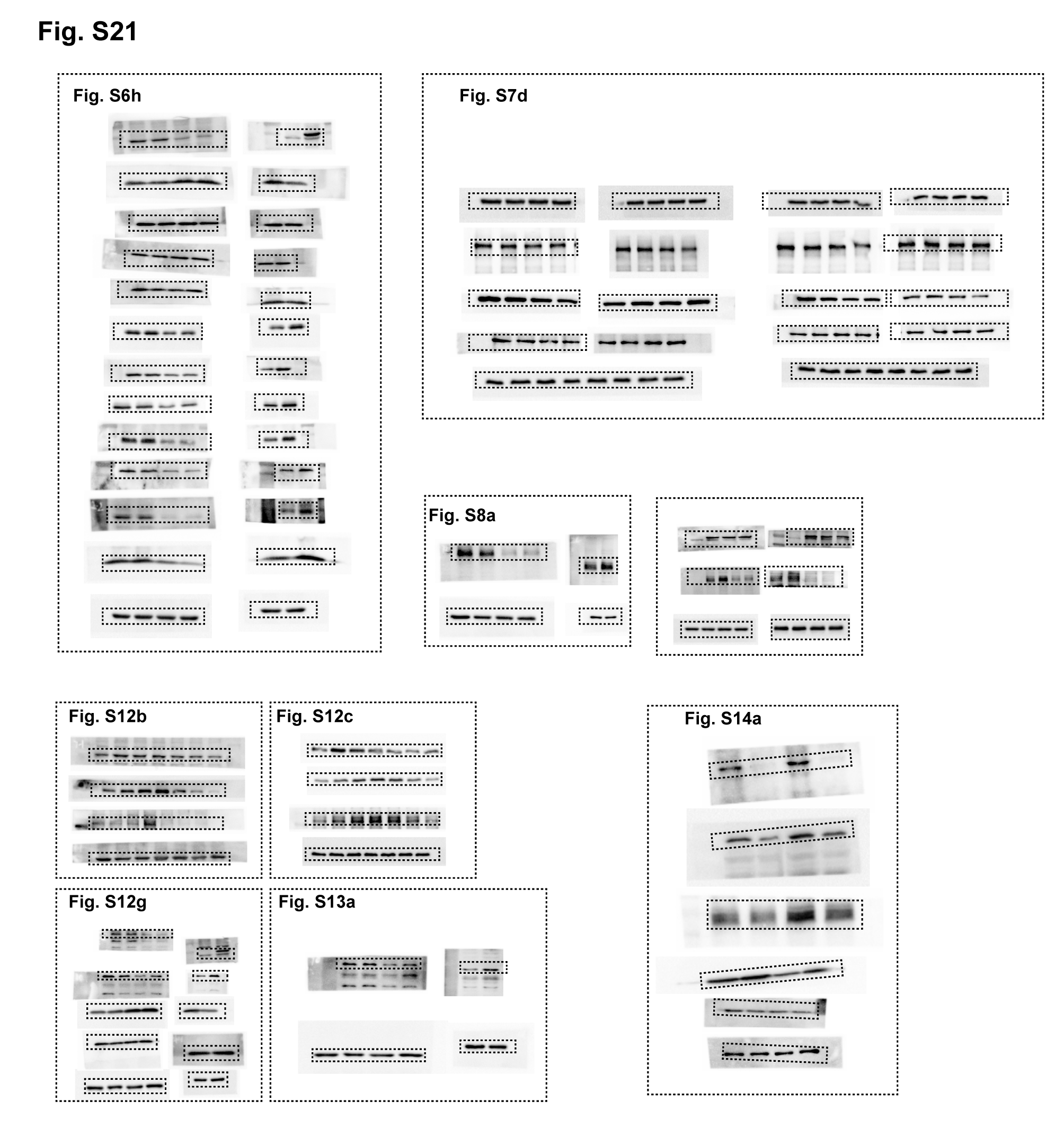


**Supplementary Fig. S21. The original and uncropped films of Western blots.**

Supplementary tables are provided as separate MS Excel spreadsheet documents.

**Supplementary Table S1.** Information of differential expressed gene from the RNA-seq analysis.

**Supplementary Table S2.** Information of differential expressed genes of livers at 36h after PH from the RNA-seq analysis.

**Supplementary Table S3.** Gene expression of cell cycle related genes from the RNA-seq analysis.

**Supplementary Table S4.** The target sequences of siRNAs used in this study.

**Supplementary Table S5.** Primers used in this study.
